# Magneto-photo-acoustic Nanotheranostics Orchestrate Ferroptosis–Immune Cross Talk for Spatiotemporally Amplified Triple-Negative Breast Cancer Therapy

**DOI:** 10.34133/bmr.0258

**Published:** 2025-10-09

**Authors:** Yibo Qiu, Huan Wu, Zijing Lin, Jieqi Chen, Shiqi Tian, Zhigang Wang, Haitao Ran, Yingxiong Wang, Long Cheng

**Affiliations:** ^1^ School of Basic Medical Sciences, Chongqing Medical University, Chongqing 400016, People’s Republic of China.; ^2^ Chongqing Key Laboratory of Ultrasound Molecular Imaging and Therapy, The Second Affiliated Hospital of Chongqing Medical University, Chongqing 400010, People’s Republic of China.; ^3^ Department of Obstetrics and Gynecology, The Second Affiliated Hospital of Chongqing Medical University, Chongqing 400010, People’s Republic of China.; ^4^ Department of Breast and Thyroid Surgery, The Second Affiliated Hospital of Chongqing Medical University, Chongqing 400010, People’s Republic of China.; ^5^ Department of Breast, Thyroid and Hernia Surgery, Yongchuan District People’s Hospital, Chongqing City, Chongqing 402160, People’s Republic of China.; ^6^ Department of General Medicine, The Affiliate Yongchuan Hospital of Chongqing Medical University, Chongqing 402160, People’s Republic of China.; ^7^ Department of Ultrasound, The Second Affiliated Hospital of Chongqing Medical University, Chongqing 400010, People’s Republic of China.

## Abstract

Triple-negative breast cancer (TNBC) remains a formidable clinical challenge owing to its aggressive behavior, immunosuppressive tumor microenvironment, and lack of effective targeted therapies. To address these limitations, we developed a magneto-photo-acoustic responsive nanoplatform (MnFe_2_O_4_–erastin–perfluoropentane nanoparticles [MEPNPs]). This nanoplatform features 3-tiered therapeutic innovations: (a) Multimodal imaging-guided precision therapy: The superparamagnetic property of MnFe_2_O_4_ enabled magnetic resonance and photoacoustic imaging, allowing real-time visualization of tumor margins. (b) Spatiotemporally controlled ferroptosis activation: Magnetic targeting enhanced the tumor accumulation of MEPNPs, while near-infrared irradiation triggered perfluoropentane vaporization for burst erastin release. This dual strategy combinationally suppressed glutathione peroxidase 4 and amplified the accumulation of lipid peroxides, achieving the amplification of ferroptosis. (c) Immunogenic tumor microenvironment reprogramming: MEPNP-induced immunogenic cell death promoted dendritic cell maturation and CD8^+^ T-cell infiltration, effectively converting immunologically “cold” TNBC tumors into “hot” phenotypes. In TNBC models, MEPNP treatment elicited remarkable therapeutic outcomes: primary tumor suppression, reduction in lung metastasis, and an extended median survival period exceeding 45 d. The transcriptome sequencing results showed that there were 6,198 differentially expressed genes in the treatment group. These included the up-regulation of ferroptosis drivers such as SLC39A14, as well as the down-regulation of antioxidant regulators such as SLC7A11 and SLC3A2. Additionally, Kyoto Encyclopedia of Genes and Genomes pathway analysis confirmed that the “ferroptosis” and “T-cell differentiation” pathways were specifically activated. This work establishes a novel “theranostic–immunomodulatory” paradigm that integrates magnetic targeting, ferroptosis potentiation, and immunogenic-cell-death-mediated immune memory. By orchestrating physical energy conversion, MEPNPs provide a spatially focused and immunologically amplified strategy to overcome TNBC therapeutic resistance.

## Introduction

Triple-negative breast cancer (TNBC), known for its aggressive behavior, immunosuppressive microenvironment, and lack of targeted treatment, is a marked clinical challenge with poor prognosis and high recurrence rates [1,2]. Current therapeutic options, such as chemotherapy and immunotherapy, are often hampered by inadequate drug delivery efficiency, off-target toxicity, and an immunosuppressive “cold” tumor phenotype [3]. Recent successes in ferroptosis induction, a unique iron-dependent cell death mechanism caused by lipid peroxidation, have demonstrated potential in inhibiting chemoresistance and reshaping the tumor microenvironment [4,5]. However, the clinical translation of ferroptosis-based treatment is limited by tumor heterogeneity, glutathione (GSH)-mediated antioxidant defense systems, and the inability to spatiotemporally modulate treatment activation [6,7].

Evolving nanotheranostic platforms that combine multimodal imaging and stimuli-responsive drug release represent a paradigm shift in TNBC treatment [8]. For example, titanium-based metal–organic frameworks with narrow bandgaps and oxygen vacancies promote photodynamic reactive oxygen species (ROS) generation, yet their limited tumor targeting and single-modality action hinder treatment efficacy [9]. Similarly, metal–phenolic coordination nanoparticles use tumor-microenvironment-responsive degradation for combinational chemodynamic/sonodynamic treatment [10]. However, they lack real-time imaging guidance and immune activation properties. Meanwhile, glutathione peroxidase 4 (GPX4) inhibitors combined with immune checkpoint blockers have shown increased T-cell infiltration in the luminal androgen receptor subtype of TNBC [11]. However, systemic toxicity and poor biodistribution persist. These shortcomings underscore the urgent need for a multifunctional nanoplatform that combines accurate targeting, multimodal imaging, ferroptosis–immune synergy, and spatiotemporal control.

A multifunctional nanoplatform requires a combination of multiple materials, posing a significant hindrance to clinical translation [12]. Fortunately, we have discovered a unique material with integrated multifunctional properties, i.e., ultrasmall manganese ferrite (MnFe_2_O_4_). MnFe_2_O_4_ is a magnetic nanomaterial with a spinel structure and an under 5-nm particle size [13]. The d-orbital electron transition of Mn^2+^/Fe^3+^ in the spinel structure provides a strong near-infrared (NIR) absorption capacity, whereas superparamagnetism allows accurate manipulation under an external magnetic field (MF) [14]. The ultrasmall size provides abundant surface-active sites, promoting Fenton-like reactions and improving the production efficiency of ROS [15]. In addition, ultrasmall manganese ferrite nanoparticles exhibit unique magnetic resonance (MR) T1 contrast effects; especially when their size is 3 nm, they show enhanced *r*_1_ relaxation rates (10.35 mM^−1^ s^−1^) [13,16]. Under NIR excitation, MnFe_2_O_4_ produces a strong photoacoustic (PA) signal via nonradiative relaxation, allowing real-time dynamic monitoring of tumor angiogenesis and drug distribution [17]. The narrow bandgap (~1.2 eV) and local surface plasmon resonance effect make its photothermal conversion efficiency reach 48.7%, hence achieving efficient local ablation of tumors [18]. Moreover, the lipid peroxidation storm induced by MnFe_2_O_4_ during the Fenton reaction can consume GSH, hence suppressing GPX4 from reducing lipid peroxides; this process successfully triggers ferroptosis in tumor cells [19,20]. This study leverages the multiphysical field coupling—integrating magnetic, photothermal, and catalytic effects—in ultrasmall manganese ferrite to overcome the limitation of single functionality in classical nanomaterials. This is geared toward providing an integrated solution of “diagnosis navigation–in situ treatment–immune enhancement” for accurate tumor diagnosis and treatment. Its “one material, multiple functions” feature simplifies the production process of nanopreparations and provides an ideal tool for customized medicine and multimechanism combinational treatment. Nonetheless, improving the biocompatibility of MnFe_2_O_4_ is another hindrance in application or clinical transformation in vivo [21].

**Fig. 1.**
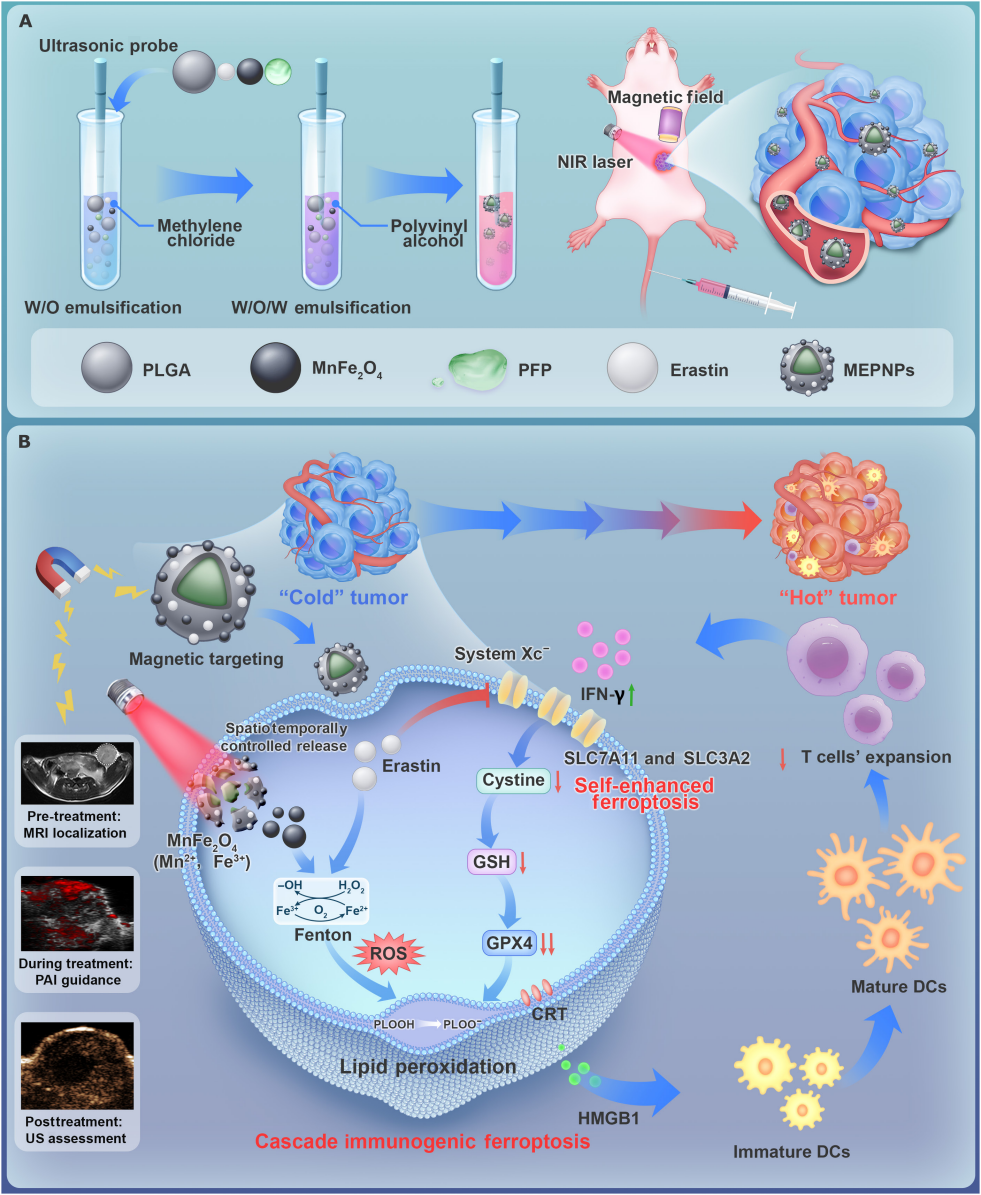
(A) The preparation process of MnFe_2_O_4_–erastin–perfluoropentane nanoparticles (MEPNPs). (B) A schematic diagram illustrating the induction of ferroptosis and immunogenic cell death (ICD) in tumor cells by MEPNPs under the magneto-photo-acoustic cascade effect: The nanoplatform of MEPNPs induces the ICD of tumor cells through ferroptosis and photothermal therapy, and the activated CD8^+^ T cells secrete interferon-γ (IFN-γ) after ICD, which further inhibits the system Xc^−^ transporter by down-regulating the SLC7A11 and SLC3A2 genes, achieving a self-enhancement process of ferroptosis. W/O, water/oil; W/O/W, water/oil/water; NIR, near-infrared; PLGA, polylactic-*co*-glycolic acid; PFP, perfluoropentane; PAI, photoacoustic imaging; US, ultrasound; GSH, glutathione; GPX4, glutathione peroxidase 4; ROS, reactive oxygen species; CRT, calreticulin; HMGB1, high mobility group box 1; DCs, dendritic cells.

We developed a magneto-photo-acoustic multimodal-responsive nanotheranostic system (MnFe_2_O_4_–erastin–perfluoropentane nanoparticles [MEPNPs]) to solve these shortcomings (Fig. 1A). The term “magneto-photo-acoustic” specifically denotes the sequential multimodal functionality: magneto, MF-guided tumor targeting and T1-weighted magnetic resonance imaging (MRI) contrast enhancement; photo, laser-triggered perfluoropentane (PFP) vaporization for PA signal generation; and acoustic, ultrasound (US)-monitored phase transition and drug release kinetics. This integrated approach enables real-time therapy guidance unattainable by single-modality systems. To improve biocompatibility, MEPNPs are developed with polylactic-*co*-glycolic acid (PLGA) as the framework, with MnFe_2_O_4_ and the ferroptosis inducer erastin loaded on the hydrophobic shell and PFP, with the capacity of liquid–gas phase transition, encapsulated in the hydrophilic core. Unlike the existing strategies, MEPNPs uniquely combine the following: (a) MR/PA/US multimodal imaging for real-time visualization of TNBC boundaries, leveraging the superparamagnetic core and phase-transitional polymer shell for dynamic lesion monitoring. (b) Spatiotemporally controlled ferroptosis activation: NIR laser-triggered liquid–gas phase transition enables on-demand release of ferroptosis inducers (erastin) and mild photothermal therapy (MPTT, Δ*T* < 45 °C), combinatorially depleting GPX4 and amplifying lipid peroxidation cascades. (c) Immune microenvironment reprogramming: By causing immunogenic cell death (ICD), MEPNPs promote dendritic cell (DC) maturation and CD8^+^ T-cell infiltration, effectively converting “cold” tumors into immunologically “hot” phenotypes.

This work represents the first combination of magneto-photo-acoustic guidance, ferroptosis–immune cross talk, and thermoresponsive drug release into a single nanoplatform (Fig. 1B). We found that the suppression rate of the primary tumor reached 78.08% ± 3.25%, and the number of lung metastases was significantly reduced. The performance was superior to that of conventional single treatment. By bridging the gap between ferroptosis induction and immune activation, MEPNPs establish a new “theranostic–immunomodulatory” paradigm, providing transformative potential for TNBC and other solid tumors resistant to current modalities. This work improves TNBC precision treatment and redefines the role of nanotheranostics in oncology by harmonizing diagnostic precision, treatment efficacy, and immune potentiation.

## Materials and Methods

### Materials

The following reagents were used in this study: PLGA (50:50, Mw = 8 kDa) and oleic acid-coated MnFe_2_O_4_ (3 nm, 8 mg/ml) were purchased from Xi’an Ruixi Biotechnology, PFP was purchased from the Nuclear Industry Physical and Chemical Engineering Research Institute (Tianjin, China), and erastin and polyvinyl alcohol (Mw = 25 kDa) were bought from Sigma-Aldrich, China. The Cell Counting Kit-8 (CCK-8) kit was from MedChemExpress (Shanghai, China). Chloroform and isopropanol were purchased from Chuandong Chemical (Chongqing, China). The oxidative-stress-related kits (malondialdehyde [MDA], GSH, and oxidized glutathione [GSSG]) were from Beyotime Biotechnology (Shanghai, China). Immunodetection assay was performed using the following reagents: anti-programmed cell death ligand 1 monoclonal antibody purchased from BioXcell, CD series flow cytometry (FCM) antibodies (CD3–fluorescein isothiocyanate [FITC], CD4–phycoerythrin [PE], CD11c–FITC, etc.) were purchased from Dakewe Biotech (Beijing, China), and mouse enzyme-linked immunosorbent assay (ELISA) kits for interleukin-12 (IL-12), tumor necrosis factor α (TNF-α), and interferon-γ (IFN-γ) were bought from Aifang Biotech (Hunan, China).

### Preparation of MEPNPs

MEPNPs loaded with MnFe_2_O_4_, erastin, and PFP were prepared using a water/oil/water double emulsion method. Briefly, MnFe_2_O_4_ (250 μl, 8 mg/ml), erastin (1 mg), PFP (100 μl), and PLGA (25 mg) were dissolved in chloroform (2 ml). The mixture was sonicated (35 W, 2 min) under ice-cold conditions to prepare a primary emulsion. Polyvinyl alcohol (5 ml, 4% w/v) was then introduced into the mixture, which underwent a second round of sonication (30 W, 2 min) to form a secondary emulsion. This emulsion was subsequently stirred magnetically with isopropanol (8 ml, 2% w/v) for 2 h. The MEPNPs were then isolated by centrifugation (10,000 rpm, 6 min) and stored at 4 °C for later use. Nanoparticles with different cargoes or fluorescent labels were prepared using the same protocol.

### Encapsulation efficiency and loading content

Standard curves for MnFe_2_O_4_ and erastin were established for the subsequent quantification. The MnFe_2_O_4_ content was measured by inductively coupled plasma mass spectrometry, and the concentration of erastin was measured utilizing high-performance liquid chromatography (HPLC). Encapsulation efficiency (EE) and loading content (LC) were calculated using the following equations: EE = (amount of loaded drug/initial drug amount) × 100%; LC = (amount of loaded drug/total nanoparticle weight) × 100%.

### Physicochemical characterization

The morphology and structure of MEPNPs were examined using transmission electron microscopy (TEM). In addition, elemental mapping and energy-dispersive x-ray spectroscopy were conducted to verify MnFe_2_O_4_ incorporation. Dynamic light scattering and zeta potential measurements were performed to determine the particle size distribution and surface charge. Their magnetic properties were analyzed using a vibrating-sample magnetometer, and magnetic targeting was examined under an external MF.

### Photothermal conversion efficiency

The photothermal conversion efficiency (*η*) was calculated using the following formula: *η* = (*H* × Δ*T*)/(*I*_0_ × *A*); *H* indicates the heat transfer coefficient, Δ*T* represents the temperature change, *I*_0_ is the laser power, and *A* indicates the absorbance [22–24]. The efficiency was validated via 808-nm laser irradiation (1.0 W/cm^2^, 5 min). Different concentrations of MEPNPs (0.5 to 4.0 mg/ml) were irradiated with an 808-nm laser (1.0 W/cm^2^, 5 min). Temperature changes were monitored using an infrared thermal camera. Photothermal stability was analyzed through 5 on/off laser cycles.

### Controlled drug release

The MEPNPs (0.2 ml, 2.0 mg/ml) were irradiated with an 808-nm laser (1.0 W/cm^2^), and the formation of microbubbles was examined under an optical microscope at different time points. The drug release profiles were analyzed using a dialysis method. MEPNPs (5 ml, 2.0 mg/ml) were sealed in regenerated cellulose dialysis bags (molecular weight cutoff: 1 kDa) and immersed in phosphate-buffered saline (PBS; pH 7.4, 0.18 M, 5% sodium dodecyl sulfate) at 37 °C with shaking. Laser-triggered release was induced by 808-nm irradiation (1.0 W/cm^2^, 5 min), and the samples were collected at 0, 1, 2, 4, 8, 12, 24, and 48 h, and erastin release was quantified by HPLC.

### Safety evaluation

#### In vitro

4T1 cells were incubated with different concentrations of MEPNPs (0.5 to 8.0 mg/ml) for 24 h, and cell viability was measured using the CCK-8 assay.

#### In vivo

BALB/c mice were intravenously injected with MEPNPs (4.0 mg/ml, 200 μl). Blood parameters, serum biochemistry, and histopathology of major organs were analyzed at 1, 3, 7, 14, and 28 d postinjection.

### Cell culture and orthotopic TNBC model

Murine TNBC cells (4T1 cells) were cultured in RPMI 1640 medium enriched with 10% fetal bovine serum at 37 °C in a 5% CO_2_ incubator. Female BALB/c mice (6 weeks old) were injected with 4T1 cells (1.2 × 10^6^ cells/mouse) into the right mammary fat pad. The growth of tumor was monitored until the volume reached ~100 mm^3^.

### Magnetic targeting

#### In vitro

4T1 cells were incubated with 1,1′-dioctadecyl-3,3,3′,3′-tetramethylindocarbocyanine perchlorate-labeled MEPNPs (1.0 mg/ml PLGA equivalent) in the presence or absence of an external MF. FCM was utilized to assess cellular uptake, and cellular uptake was visualized through confocal microscopy.

#### In vivo

1,1-Dioctadecyl-3,3,3,3-tetramethylindotricarbocyanine iodide (DiR)-labeled MEPNPs (2.0 mg/ml, 200 μl) were intravenously administered into tumor-bearing mice. A neodymium magnet (341 mT) was placed near the tumor for 4 h. Fluorescence imaging was performed at 0.5, 1, 2, 3, 4, and 5 h postinjection.

### Multimodal imaging

#### In vitro

MRI: T1-weighted imaging (repetition time/echo time = 500/10 ms) was conducted to explore the T1 signal intensity. Photoacoustic imaging (PAI): MEPNPs were scanned at 680 to 970 nm to determine the optimal excitation wavelength. US: Echo intensity was measured before and after laser irradiation (808 nm, 1.0 W/cm^2^).

#### In vivo

MRI: T1-weighted imaging (repetition time/echo time = 650/11 ms) was employed to calculate the signal enhancement percentage (PSIE). PAI: Tumor PA signals were quantified at 1, 2, 3, 4, and 5 h postinjection. US: Changes in echo intensity were analyzed following laser irradiation.

### Oxidative stress evaluation

The concentration of GSH, GSSG, and MDA in 4T1 cells was measured using corresponding commercial kits. ROS and lipid peroxidation were measured using 2′,7′-dichlorodihydrofluorescein diacetate (DCFH-DA) and boron dipyrromethenes (BODIPY) 581/591 C11 probes, respectively. The mitochondrial membrane potential was determined through JC-1 staining assay. 4T1 cells treated with MEPNPs were fixed with 2.5% glutaraldehyde, and mitochondrial morphology was examined using TEM.

### Transcriptome sequencing

RNA sequencing was performed by Qiantang Life Science and Technology Co., Ltd (Suzhou, China). Quality control of the sequenced data and construction of the genome map were completed using the Fastp software to ensure the quality and reliability of data analysis. Significant differences were based on the following thresholds: corrected *P* value < 0.05 and |log_2_(fold change)| > 1. Gene Ontology (GO) term and pathway enrichment analyses were conducted using the R package clusterProfiler based on the GO and Kyoto Encyclopedia of Genes and Genomes (KEGG) databases. GO terms or KEGG pathways with *P* < 0.05 were considered significantly enriched. Ferroptosis-related genes were screened from the FerrDb database, and ICD-related genes were obtained from the National Center for Biotechnology Information database.

### In vitro immune activation

A Transwell system was used to assess the in vitro activation of DCs. Initially, 4T1 cells were inoculated in the upper chamber, while immature DCs were seeded in the lower chamber. The samples were randomly divided into the control group (PBS), erastin–PFP nanoparticles (EPNPs) group, MnFe_2_O_4_–PFP nanoparticles (MPNPs) + MF + L (laser) group, and MEPNPs + MF + L group and incubated with the corresponding treatments. Subsequently, 4T1 cells in the upper chamber were collected and co-cultured in the lower chamber for 24 h. Finally, the DCs were collected and subjected to FCM analysis using anti-CD11c–FITC, anti-CD86–PE and anti-CD80–allophycocyanin antibodies (eBioscience, Thermo Scientific USA). The supernatants were collected for the detection of IL-12 and TNF-α using their corresponding ELISA kits.

### Therapeutic efficacy

#### In vitro

4T1 cells were treated with MEPNPs (2.0 mg/ml) and irradiated with an 808-nm laser (1.0 W/cm^2^, 5 min). Next, cell viability was investigated using the CCK-8 assay, and apoptosis was quantified by FCM.

#### In vivo

All tumor-bearing mice were randomly divided into 6 groups: the control group, laser-only group, EPNPs group, MEPNPs + L group, MPNPs + MF + L group, and MEPNPs + MF + L group (*n* = 5). On days 0, 3, and 6, the mice were injected with the corresponding nanoparticles via the tail vein (concentration: 2.0 mg/ml, 200 μl). For the groups with external MFs, circular magnets of the same magnetic force were placed on the tumor surface immediately after injection and fixed with adhesive tape. The magnets were removed after 4 h, and the tumor area was irradiated with an 808-nm laser (1.0 W/cm^2^, 5 min). The temperature changes in the tumor area were monitored using an infrared thermal imager. On day 9, one mouse from each group was sacrificed through cervical dislocation and major organs (heart, liver, spleen, lung, and kidney) were subjected to the hematoxylin and eosin (H&E) staining assay and tumor tissues were examined by terminal deoxynucleotidyl transferase dUTP nick end labeling (TUNEL), Ki-67, and H&E staining. The expression levels of GPX4 and cyclooxygenase-2 (COX2) proteins in tumor tissues were detected by Western blotting. The content of MDA in tumor tissues was measured using the MDA kit. The body weight and tumor volume of the mice were recorded throughout the treatment period. The tumor volume was calculated using the following formula: *V* = *LW*^2^/2 (*L*, tumor length; *W*, tumor width). The relative tumor volume was calculated as *V*/*V*_0_ (*V*_0_, tumor volume before treatment). On day 14 after treatment, the mice were euthanized, and the tumor tissues were removed and weighed. The tumor growth inhibition rate was calculated as (1 − tumor weight in the treatment group/tumor weight in the control group) × 100%.

### In vivo immune activation

To investigate whether photothermal therapy (PTT) combined with ferroptosis could enhance anti-tumor immunity and prevent distant metastasis in vivo, tumor-bearing mice were randomly divided into 6 new groups: the control group, laser-only group, EPNPs group, MEPNPs + L group, MPNPs + MF + L group, and MEPNPs + MF + L group (*n* = 5). Mice in each group were intravenously injected with the corresponding drugs via the tail vein on days 0, 3, and 6. Both the MPNPs + MF + L group and the MEPNPs + MF + L group required the concurrent application of an external MF and laser irradiation. On day 9, FCM was used to assess the proportions of DC maturation and cytotoxic T lymphocyte infiltration in tumor tissues, lymph nodes, and the spleen. Immunofluorescence staining was performed on tumor tissues (calreticulin [CRT], high mobility group box 1 [HMGB1], heat shock protein 70 [HSP70], CD8, and IFN-γ). Meanwhile, the concentrations of IL-12, TNF-α, and IFN-γ in the blood were detected using their respective ELISA kits.

### Lung metastasis model

4T1 cells in the logarithmic growth phase were collected to ensure optimal viability and proliferative capacity. Following enzymatic digestion and centrifugation, the cells were resuspended in PBS and thoroughly mixed. During cell suspension preparation, care was taken to avoid excessive cell aggregation or uneven dispersion. Aggregated cell clusters can cause pulmonary embolism in mice during modeling. Next, 6-week-old BALB/c female mice were chosen and disinfected at the puncture point on the tail with an alcohol cotton ball. The prepared cell suspension was administered into the mouse via the tail vein. The number of cells injected into each mouse was 2 × 10^5^ [25]. The health status and survival of the mice daily after injection was monitored.

### Evaluation of the effect of inhibiting metastasis

To examine the effect of intracellular nanoparticles on the distant metastasis of TNBC, tumor-bearing mice were randomly divided into 6 groups: the control group, laser-only group, EPNPs group, MEPNPs + L group, MPNPs + MF + L group, and MEPNPs + MF + L group (*n* = 5). Mice in each group were injected with the corresponding drugs via the tail vein on days 0, 3, and 6. The MPNPs + MF + L group and MEPNPs + MF + L group received simultaneous application of an external MF and laser intervention. On day 10, the 4T1 cell suspension (2 × 10^5^ cells per mouse) was administered via the tail vein to promote lung metastasis. The survival of the mice was closely monitored thereafter. On day 45, the mice were sacrificed, and their lungs were dissected and examined for metastasis. The anti-metastatic efficacy of the nanoparticles was assessed by examining the number, size, and distribution of metastatic nodules on the lung surface. Lung tissues were fixed in 10% formalin for 24 h, embedded in paraffin, sectioned (5 mm), and stained with H&E for histological assessment of metastatic nodules.

### Statistical analysis

The measurement data are expressed as mean ± standard deviation, and all statistical analyses were performed using the SPSS 27.0 software. The *t* test was used to compare significant differences between 2 groups, and analysis of variance was used to compare multiple groups. Survival data were analyzed using the log-rank test in GraphPad Prism 9.0, with *P* < 0.05 considered statistically significant. The significance test criteria were as follows: ns, no statistically significant difference between the 2 groups; *P < 0.05; **P < 0.01; ***P < 0.001; and ****P < 0.0001.

## Results and Discussion

### Fabrication and characterization of MEPNPs

**Fig. 2.**
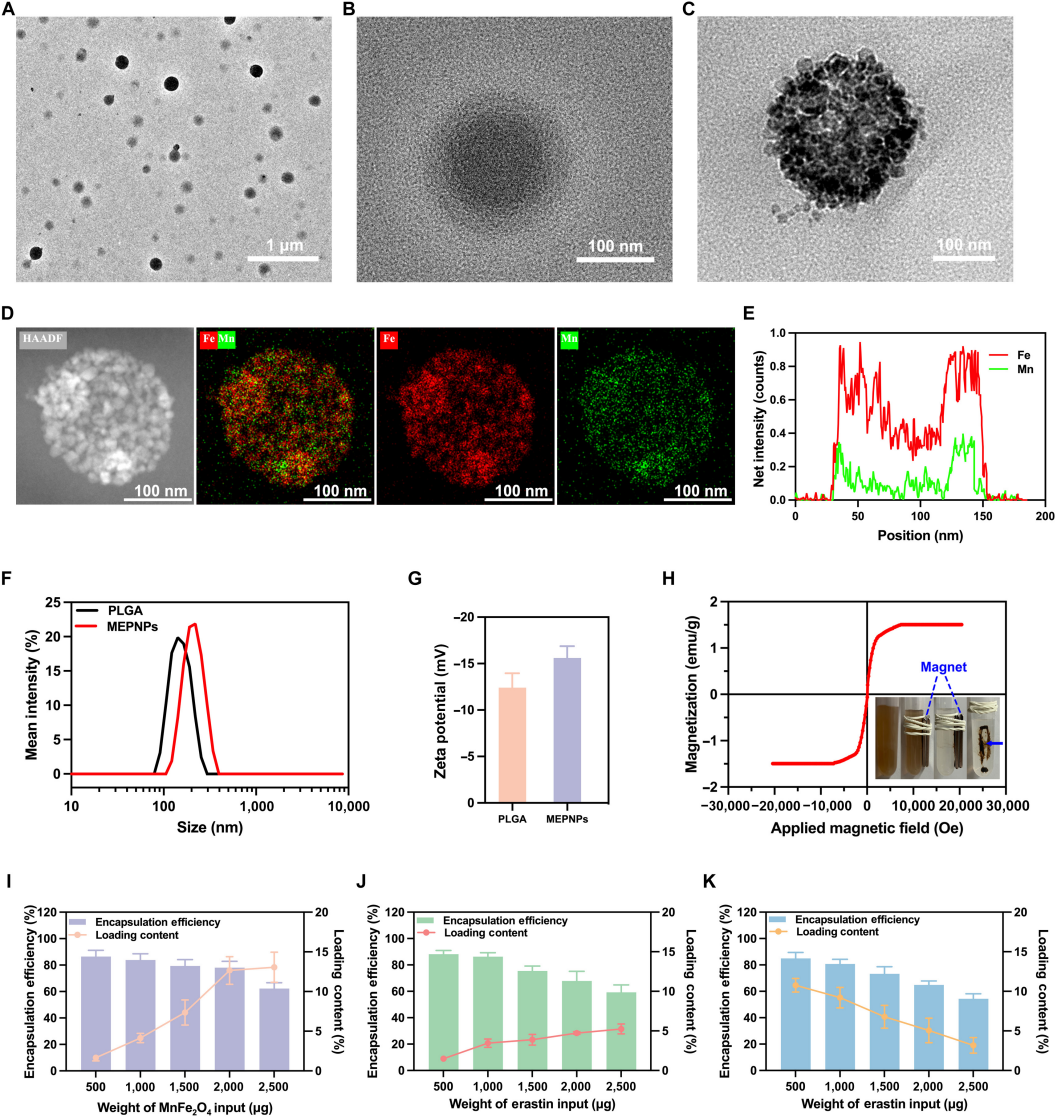
Characterization of MEPNPs. (A) Transmission electron microscopy (TEM) image of MEPNPs under a low-power microscope. (B) TEM image of erastin–PFP nanoparticles (EPNPs) under a high-power microscope. (C) TEM image of MEPNPs under a high-power microscope. (D) Element mapping of MEPNPs. (E) Element line sweeps of MEPNPs. (F) Size distribution of PLGA and MEPNPs. (G) Zeta potential of PLGA and MEPNPs. (H) The magnetization hysteresis loop of MEPNPs. (I and J) Encapsulation efficiency and loading content of MnFe_2_O_4_ and erastin at different initial input doses. (K) Encapsulation efficiency and loading content of MnFe_2_O_4_ at different initial input doses of erastin. The experiments were repeated thrice independently. HAADF, high-angle annular dark field.

MEPNPs were prepared using a typical double emulsion method (water/oil/water); i.e., lipophilic compounds (MnFe_2_O_4_ and erastin) were loaded into the PLGA shell and a hydrophilic compound (PFP) was encapsulated in the core of PLGA nanoparticles (Fig. 1A) [26]. TEM images revealed that the nanoparticles had uniform spherical and core–shell structures (Fig. 2A). Unlike EPNPs, MEPNPs had numerous deep black metallic substances on their surfaces (Fig. 2B and C). TEM observations revealed that the black small particles on the surface of MEPNPs were the direct manifestation of MnFe_2_O_4_. This is because when a transmission electron microscope uses an electron beam to penetrate the sample, elements with different atomic numbers have different abilities to scatter electrons. The heavy metal elements such as Fe and Mn in MnFe_2_O_4_ strongly scatter electrons, showing up as black spots in the TEM image, while the PLGA polymer weakly scatters electrons, presenting a light background. This contrast difference makes the MnFe_2_O_4_ particles embedded in the surface or near-surface region of PLGA nanoparticles clearly distinguishable. The presence of these particles confirms the successful loading of manganese ferrite onto the surface or near-surface area of PLGA nanoparticles, serving as direct evidence of their composite structure. Elemental mapping and line scan analysis showed Fe and Mn distribution in MEPNPs, verifying the successful loading of MnFe_2_O_4_ (Fig. 2D and E). Meanwhile, the average particle size of PLGA detected by dynamic light scattering was 192.67 ± 2.99 nm, which increased to 205.87 ± 4.29 nm after loading MnFe_2_O_4_, erastin, and PFP (Fig. 2F). Previous studies have reported that nanoparticles of this size are unlikely to be excreted through collapsed lymphatic vessels within tumors and have a strong enhanced permeability and retention (EPR) effect [27–29]. The zeta potentials of PLGA and MEPNPs were −12.40 ± 1.55 and −15.60 ± 1.28 mV, respectively (Fig. 2G). Research reports indicate that the surface charge of nanoparticles is one of the key factors influencing their circulation time in the bloodstream [30,31]. Positively charged nanoparticles tend to adsorb negatively charged plasma proteins, forming a “protein corona”, which makes them more easily recognized and cleared by macrophages, resulting in a shortened circulation time. In contrast, neutral or negatively charged nanoparticles reduce the adsorption of plasma proteins and lower the clearance rate by the reticuloendothelial system (RES), thereby prolonging their circulation time. The magnetization hysteresis loop outcomes showed that MEPNPs had no apparent remanent magnetization, indicating a clear paramagnetic property, and rapid aggregation of nanoparticles in the area with an external MF (Fig. 2H). This confirms the successful encapsulation of MnFe_2_O_4_ in MEPNPs and indicates that MEPNPs are potentially effective for MRI and magnetic targeting. The above findings collectively demonstrate that MEPNPs were successfully synthesized and had the expected morphological, physical, and chemical features, allowing for further in vitro and in vivo performance research.

To improve the performance of MEPNPs, we computed the EE and LC of MnFe_2_O_4_ and erastin at different initial input doses based on the standard curve and optimized the synthesis of MEPNPs (Fig. S1). The LC increased with increasing initial input dose of MnFe_2_O_4_. However, the EE dropped sharply and the LC reached a plateau when the input dose of MnFe_2_O_4_ exceeded 2.0 mg (Fig. 2I). Since MnFe_2_O_4_ is the most critical component in the nanocarrier system for imaging (MRI and PAI) and treatment (MPTT and ferroptosis), we first determined the optimal input dose of MnFe_2_O_4_ (2.0 mg). Consequently, the EE and LC of erastin also increased with increasing input dose of erastin. Nevertheless, the EE and LC of MnFe_2_O_4_ decreased due to the competition for shell space between the 2 lipophilic substances (Fig. 2J and K). Eventually, we chose the initial input doses of MnFe_2_O_4_ (2.0 mg) and erastin (1.0 mg) to balance the imaging capacity and treatment effect. After the optimized synthesis, the EE and LC of MnFe_2_O_4_ in MEPNPs were 80.79% ± 2.84% and 9.20% ± 1.06%, respectively, and those of erastin were 86.20% ± 2.46% and 3.45% ± 0.43%, respectively.

### Photothermal conversion and photo-controlled drug release

To confirm the photothermal conversion performance of MEPNPs, an infrared thermal imager was used to record the temperature change process of MEPNPs at different concentrations (0, 0.5, 1.0, 2.0, and 4.0 mg/ml) and different laser powers (0.5, 1, and 1.5 W/cm^2^) (Fig. S2A to D). The time–temperature curves revealed that MEPNPs had a substantial concentration- and power-dependent photothermal effect. This suggests that the heating effect of MEPNPs could be accurately regulated by adjusting the concentration, laser power, and irradiation duration. Meanwhile, to clearly define the specific contribution of MnFe_2_O_4_ to the thermal effect, we supplemented the temperature changes monitored by an infrared imager of different nanoparticle solutions under laser irradiation (groups: PBS + L, EPNPs + L, MPNPs + L, and MEPNPs + L). The results show that only in the presence of MnFe_2_O_4_ did the nanoparticles exhibit excellent photothermal conversion effects, indicating that MnFe_2_O_4_ is the substance that endows MEPNPs with photothermal conversion ability (Fig. S3). Since the EPNPs + L group showed no significant temperature increase in the in vitro photothermal conversion experiment after laser irradiation and in accordance with the requirements of animal ethics to minimize the number of animals used, we did not add the EPNPs + L group to the in vivo photothermal conversion observation experiment. The time–temperature curves of 5 on/off cycles revealed that the temperature peak remained at approximately 45 °C, demonstrating the excellent photothermal stability of MEPNPs (Fig. S2E). These findings lay a solid foundation for subsequent in vivo PTT. Based on the reported method, the photothermal conversion efficiency of MEPNPs was 24.1%, which was superior to most of previously reported photothermal conversion nanomedicines (Fig. S2F and G) [32–34].

After evaluating the thermally induced phase change performance of MEPNPs, we observed the number of microbubbles in MEPNPs under laser irradiation for different durations under an optical microscope (Fig. S2H). The statistical findings revealed that the number of microbubbles in MEPNPs gradually increased with the extension of laser irradiation time, reaching a peak at 4 min. The number of microbubbles observed under the microscope significantly decreased due to the continuous expansion and rupture of microbubbles (Fig. S2I). This confirms that the phase change material PFP was successfully encapsulated in the nanoparticles and demonstrates the capacity of MEPNPs to undergo thermally induced phase change upon laser irradiation.

Based on the standard curve of erastin, we computed the cumulative release of erastin at different time points after laser irradiation to evaluate the controlled drug release performance of MEPNPs. As a consequence, erastin release in the EPNPs + L group without loading MnFe_2_O_4_ and the MEPNPs group without laser irradiation was relatively slow, reaching a plateau at 24 h with a release ratio of only approximately 40%. In contrast, the MEPNPs + L group had a release rate of over 80% within the first 12 h before entering a plateau, with an average release ratio of 85.99% ± 3.10% at 24 h (Fig. S2J). This shows that although MEPNPs had a slow drug release capacity by themselves, laser irradiation could rapidly increase the drug release to a peak. Due to the inherent hydrolysis and enzymatic degradation of the PLGA polymer matrix over time, a sustained and slow release of the encapsulated drugs is achieved. Meanwhile, the mechanical force generated during the thermally induced phase transition prompts the formation of transient pores or even rupture of the nanoparticles, which triggers an instantaneous burst release of the encapsulated drugs. The baseline release not only ensures a certain drug concentration within the tumor before the burst release but also allows the nanoparticles that have not undergone phase transition to continue the slow release of drugs after the burst release, thereby counteracting the natural clearance effect in the body and maintaining a high-drug-concentration environment for a relatively long time. The combination of these 2 release mechanisms can overcome the limitations of drug diffusion and clearance effects in the body. Therefore, the combination of sustained release and burst release is conducive to rapidly achieving and maintaining an effective drug concentration level within the tumor for a long time, achieving high-intensity killing of tumor cells.

### Magnetic targeting performance

**Fig. 3.**
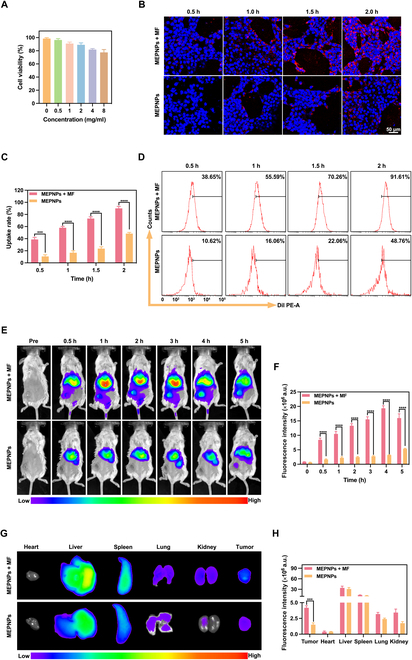
Magnetic targeting performance. (A) The Cell Counting Kit-8 (CCK-8) assay was used to detect the cell viability after the cells had been co-incubated with different concentrations of MEPNP solution. (B) Confocal laser scanning microscopy (CLSM) images of the cell uptake of MEPNPs in the presence or absence of a magnetic field. (C) Statistical chart of the proportion of nanoparticle uptake by 4T1 cells in each group (*n* = 3). (D) Flow cytometric quantification of MEPNP uptake by cells at different times. (E) In vivo fluorescence images of the distribution of MEPNPs (*n* = 3). (F) Fluorescence intensity of tumors at different time points. (G) Fluorescence images of isolated organs and tumors. (H) Fluorescence intensity of isolated organs and tumors (*n* = 3). ***P < 0.001; ****P < 0.0001. MF, magnetic field. DiI PE-A, 1,1′-dioctadecyl-3,3,3′,3′-tetramethylindocarbocyanine perchlorate phycoerythrin-A.

The biocompatibility of the nanodrug delivery platform is a prerequisite for its clinical use [35]. Therefore, the safety of MEPNPs was assessed before carrying out in vitro and in vivo studies. Firstly, the CCK-8 assay was used to determine the effect of MEPNPs on cell viability in the absence of laser irradiation. Consequently, the survival rate of 4T1 cells was still greater than 80% when the concentration of MEPNPs reached 4 mg/ml, indicating that MEPNPs have a relatively high safe concentration without laser excitation (Fig. 3A). To further explore the safety of MEPNPs in healthy BALB/c mice, blood routine tests and serum biochemical analyses were conducted on days 1, 3, 7, 14, and 28 following tail vein injection (200 μl, concentration 4.0 mg/ml) (saline injection was used as the control group). As a result, no abnormal blood indicators were observed within 28 d after MEPNP injection (Fig. S4A). Moreover, no pathological changes were observed in the H&E staining results of the major organs (heart, liver, spleen, lung, and kidney) (Fig. S4B). These findings strongly demonstrate that MEPNPs exhibit good biocompatibility and can be used for subsequent in vivo imaging and therapeutic studies.

Based on confocal laser scanning microscopy (CLSM), we observed that the red fluorescence around the blue nuclei of 4T1 cells in the MEPNPs + MF group was significantly stronger than that in the MEPNPs group without an external MF. This indicates that MEPNPs possess good magnetic targeting capacity (Fig. 3B). Moreover, CLSM images revealed that the intensity of the red fluorescence around the nuclei of 4T1 cells increased over time under the influence of the MF, showing a significant enhancement within 2 h. This acts as the basis for the duration of the MF in subsequent in vitro cell experiments. Subsequently, FCM quantitative detection revealed that the intracellular nanoparticle uptake rates of the MEPNPs + MF group and the MEPNPs group after co-incubation with cells for 2 h were 90.03% ± 2.70% and 48.33% ± 1.65%, respectively (Fig. 3C and D). Moreover, statistical analysis of the mean fluorescence intensity of each group of cell solutions indicated that the cell solutions in the MF group took up more MEPNPs (Fig. S5). These results indicate that the MF significantly enhanced the association of MEPNPs with 4T1 cells, leading to an increase in intracellular fluorescence intensity. Although this might involve improved cellular uptake, further studies in the future are still needed to distinguish between surface adsorption and internalization [36,37].

Subsequently, in vivo fluorescence imaging was carried out on tumor-bearing mice to assess the tumor-targeting capacity and tissue distribution of MEPNPs in vivo. The prepared DiR-labeled MEPNP solution was injected into the tail vein of mice. The in vivo fluorescence imaging outcomes revealed that the fluorescence signal at the tumor site in the MEPNPs + MF group was significantly stronger than that in the MEPNPs group. Moreover, the fluorescence intensity in the tumor area increased with the duration of the MF, reaching a peak at 4 h, hence providing a time basis for in vivo imaging and therapy (Fig. 3E). According to fluorescence quantitative analysis, the fluorescence intensity values of the MEPNPs + MF group were higher than those of the MEPNPs group at each time point following MEPNP injection. At 4 h after injection, the average fluorescence intensities in the tumor area of the MEPNPs + MF group and the MEPNPs group were 19.35 ± 0.89 × 10^6^ and 3.17 ± 0.12 × 10^6^, respectively (Fig. 3F). Additionally, fluorescence analysis was carried out on major tissues and organs including the heart, liver, spleen, lung, kidney, and tumor of separation. One hour after separation, the results revealed that the fluorescence signal of the tumor in the MEPNPs + MF group was still 2.8 times that of the MEPNPs group (Fig. 3G and H).

### Biodistribution and clearance profiles of MEPNPs

Comprehensive assessment of MEPNPs’ in vivo fate revealed favorable tumor-targeting and clearance characteristics. Fluorescence imaging demonstrated MF-driven tumor accumulation, with the signal intensity in the MEPNPs + MF group reaching 2.8-fold higher than that of non-MF controls at 4 h postinjection (Fig. 3E to H). Organ-level distribution (Fig. 3G) followed the expected RES dominance, liver > spleen > tumor (MF group) > kidneys, aligning with the classic hepatobiliary clearance of PLGA nanoparticles. Critically, longitudinal safety evaluation (Fig. S4) showed no abnormalities in hepatorenal function markers (alanine aminotransferase, aspartate aminotransferase, and blood urea nitrogen) or hematological parameters (red blood cells, white blood cells, and platelets) over 28 d. Histopathology (Fig. S4B) further confirmed the absence of pathological alterations in major organs, collectively attesting to progressive and safe clearance. These findings—MF-enhanced tumor targeting, RES-mediated biodistribution, and the absence of long-term toxicity—support the translational viability of the nanoplatform.

### Multimodal imaging of MRI/PAI/US

**Fig. 4.**
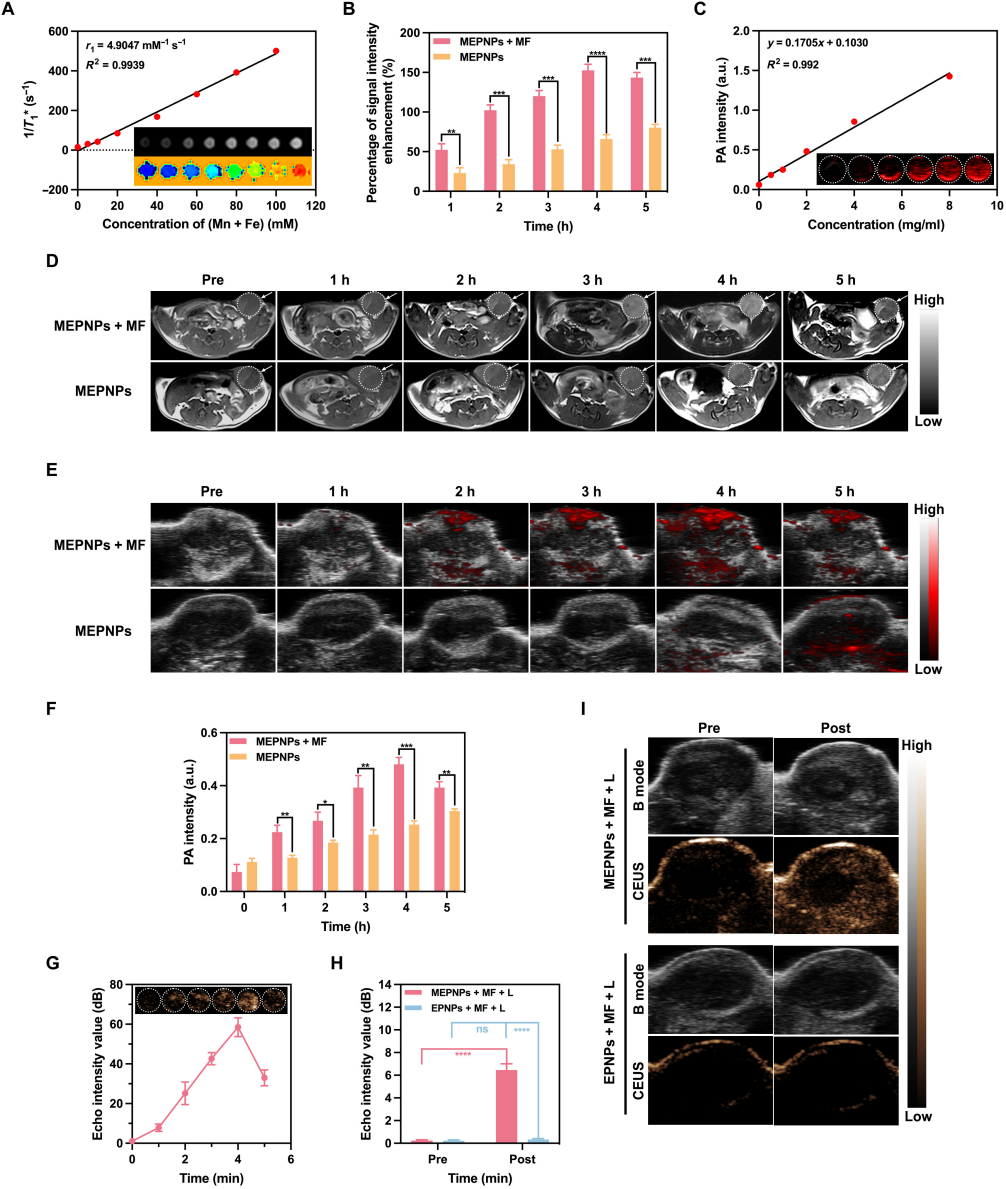
Multimodal imaging of MRI/PAI/US. (A) T1 relaxation rate of MEPNPs and the corresponding in vitro magnetic resonance (MR) images (inset). (B) The corresponding signal enhancement percentage (PSIE) of tumors (*n* = 3). (C) Linear relationship between photoacoustic (PA) intensities and MEPNP concentrations and the corresponding in vitro PA images (inset). (D) T1-weighted MR images of tumors (marked with dotted circles and arrowheads) in vivo. (E) PA images of tumors. (F) The corresponding signal intensities of PA (*n* = 3). (G) In vitro US images in the contrast-enhanced ultrasound (CEUS) of MEPNPs at varying radiation times (inset). (H) The in vivo echo intensity values in CEUS (*n* = 3). (I) In vivo US images in the CEUS of tumor. *P < 0.05; **P < 0.01; ***P < 0.001; ****P < 0.0001.

First, we evaluated the potential of MEPNPs as MRI contrast agents. Unlike the PBS of the control group, the MEPNP solution had excellent MRI T1-weighted imaging performance (Fig. 4A). As the concentration of MEPNPs increased (the molar concentrations of Mn + Fe ranges from 5 to 100 mM), the brightness of the T1-weighted images also increased. A similar trend was observed in the MR mapping images. Subsequently, the T1 relaxation time was measured at different concentrations to assess the improvement effect of MEPNPs on image contrast, thereby determining the potential of MEPNPs as an MRI T1 contrast agent. The relaxation rate (*r*_1_ value) was eventually calculated as 4.91 mM^−1^ s^−1^, demonstrating that MEPNPs require only a lower concentration than the traditional Gd agent (*r*_1_ = 3.5 to 4.5 mM^−1^ s^−1^) to achieve a similar effect. Based on this, the T1-weighted imaging capacity of MEPNPs was further investigated in tumor-bearing mice. The findings revealed that the boundaries between the tumor area (marked with dotted circles and arrowheads) and the surrounding normal tissue in the MEPNPs + MF group were clearer, unlike the group without an MF (Fig. 4D). Moreover, the brightness of the tumor area gradually increased over time, reaching a peak at 4 h after injection, which corroborated the findings of in vivo fluorescence imaging. Using percentage of signal intensity enhanced (PSIE) quantitative analysis, the T1-weighted signal of the MEPNPs + MF group increased by 152.53% ± 7.67% at 4 h, whereas that of the group without MF increased by only 65.77% ± 5.49% (Fig. 4B). In vivo MRI outcomes confirmed that MEPNPs under MF guidance exhibit excellent T1-weighted contrast enhancement at tumor sites, suggesting their potential as an MRI contrast agent for tumor localization during magnetically targeted therapy.

Secondly, we systematically evaluated the PAI properties of MEPNPs. Through full-wavelength spectral scanning analysis, MEPNPs had broad-spectrum light absorption features within the NIR window of 680 to 970 nm and an absorption peak at 715 nm (Fig. S6). Based on this spectral feature, 715 nm was selected as the optimal excitation wavelength for subsequent PAI studies. In vitro experimental findings showed that under 715-nm excitation, the PA signal intensity of the MEPNP solution had a good linear positive correlation with the nanoparticle concentration within the range of 0.05 to 8.0 mg/ml (*y* = 0.1705*x* + 0.1030, *R*^2^ = 0.992), with a detection limit of 23 μg/ml (Fig. 4C). Based on this, a standard concentration–signal intensity calibration curve was established. Further, in vivo experiments were carried out to evaluate the tumor imaging efficacy of MEPNPs (Fig. 4E). Dynamic monitoring revealed that the PA signal intensity of the tumor area in the MEPNPs + MF experimental group increased in a time-dependent manner, reaching a peak at 4 h after MF intervention, which was 5.3 times higher than the baseline value (Fig. 4F). Notably, the signal intensity at each time point in this group was significantly higher than that of the control group without an MF (*P* < 0.05). On the other hand, the control group exhibited only a marginal increase in signal intensity throughout the observation period, in stark contrast to the rapid and substantial signal enhancement observed in the MEPNPs + MF group. This imaging dynamic feature was highly consistent with the distribution trend of nanoparticles monitored by MRI, suggesting that the MF-mediated active targeting significantly improved the enrichment efficiency of MEPNPs at the tumor site. We speculate that this enhancement effect may be attributed to the combinational effect of MF gradient-guided physical aggregation and the EPR effect of tumor tissues. This provides a key pharmacokinetic basis for subsequent targeted therapy research.

In vitro experiments eventually confirmed the photothermal-phase transition coupling characteristics of MEPNPs. Under the irradiation condition of an 808-nm NIR laser (1.0 W/cm^2^), real-time US monitoring showed that the backscattering signal intensity of the MEPNP suspension reached its peak at 4 min (58.5 ± 3.1 dB), before showing a significant attenuation with the extension of irradiation time (Fig. 4G). Thermodynamic analysis revealed that the corresponding system temperature at this time point (44.7 ± 2.3 °C) significantly exceeded the phase transition threshold of PFP (29 °C). This suggests that excessive heat accumulation induced the disintegration of the nanocarrier structure, causing continuous rupture of microbubbles generated by the liquid–gas phase transition. This process is highly consistent with the bubble generation and collapse process recorded under the optical microscope. Based on the spatiotemporal pharmacokinetic analysis using multimodal imaging (fluorescence, MRI, and PAI), the tumor-targeted enrichment window period of MEPNPs under MF guidance was 4 h after intravenous injection (this time window also corresponded to the duration of MF intervention). Thus, we removed the MF device 4 h after administering the nanoformulation, strictly based on the laser parameters determined in vitro (1.0 W/cm^2^ and 4 min) to precisely carry out photothermal excitation of the tumor. Real-time US contrast imaging showed that the MEPNPs + MF + L group presented a significantly enhanced echo intensity immediately after irradiation (peak 6.46 ± 0.74 dB vs. baseline 0.24 ± 0.02 dB, *P* < 0.001) (Fig. 4H and I). On the other hand, the MENPs control group without phase transition materials did not show significant signal changes (0.23 ± 0.01 dB vs. 0.34 ± 0.03 dB, *P* = 0.37). This phenomenon revealed that the PFP encapsulated in MEPNPs could achieve on-demand triggered in situ generation of microbubbles via the photothermal-phase transition combinational mechanism. Specifically, after converting the laser into heat energy through the MEPNP nanocapsule layer, the liquid–gas phase transition of PFP was stimulated to generate enhanced US contrast signals. As supported by extensive prior research, the cavitation effect generated by microbubble collapse promotes reversible opening of endothelial intercellular junctions in tumor blood vessels, facilitating enhanced nanoparticle extravasation [38–41]. This dual physical response mechanism provides an innovative strategy for spatiotemporal controlled drug release.

The MRI/PAI/US multimodal imaging performance of MEPNPs spans the entire treatment process, facilitating precise treatment. Pre-treatment diagnosis and localization: MRI is used for sensitive diagnosis and precise localization of tumors before treatment, providing guidance for treatment. Real-time dynamic guidance during treatment: PAI is employed during treatment to guide the position of laser irradiation in real time and adjust the laser irradiation power and time based on tumor size. Posttreatment assessment: US is immediately used after treatment to evaluate the microbubble generation and drug release due to the thermal phase transition of MEPNPs, and the therapeutic effect is assessed through tumor blood flow signals.

### Multigenomics-based analysis of therapeutic mechanisms

**Fig. 5.**
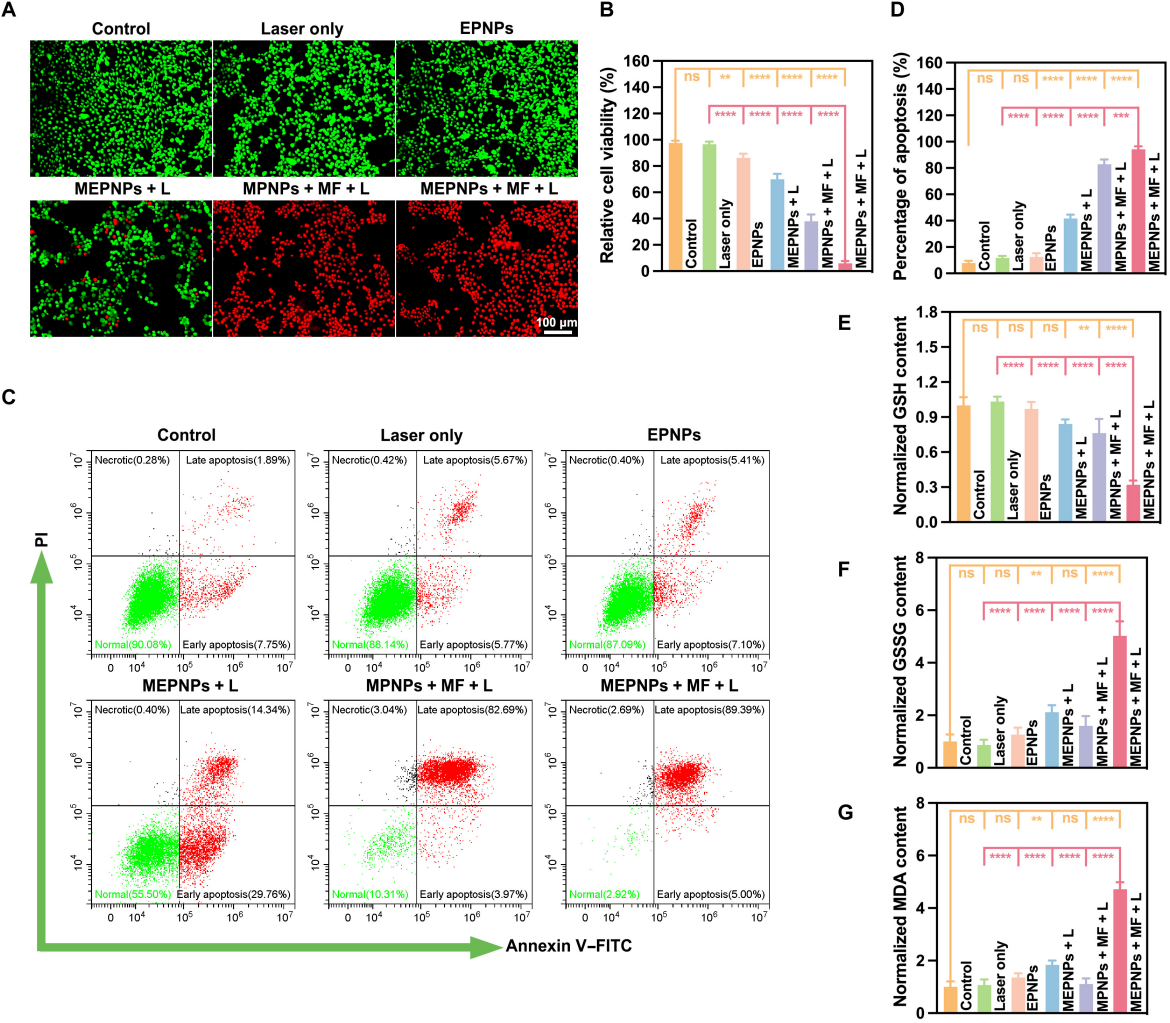
In vitro therapeutic effects of MEPNPs. (A) Confocal laser scanning microscopy (CLSM) images of 4T1 cells co-stained with calcein-AM (CAM) and propidium iodide (PI) after various treatments to distinguish the live (green fluorescence) and dead (red fluorescence) cells. (B) CCK-8 results after various treatments (*n* = 3). (C) Flow cytometry results after various treatments. (D) Statistical analysis of the apoptotic ratios of each group. The normalized GSH (E), oxidized glutathione (GSSG) (F), and malondialdehyde (MDA) (G) contents in 4T1 cells after different treatments. **P < 0.01; ***P < 0.001; ****P < 0.0001. FITC, fluorescein isothiocyanate. MPNPs, MnFe_2_O_4_–PFP nanoparticles.

Based on the cytotoxicity assessment using the CCK-8 assay, simple NIR laser irradiation (1.0 W/cm^2^, 4 min) had no significant effect on the survival rate of 4T1 cells (96.55% ± 1.67% vs. 97.56% ± 1.37% in the control group, *P* = 0.42) (Fig. 5B). This rules out the interference of the photothermal effect on the experimental system. The EPNPs treatment group showed a statistically significant decrease in cell viability due to the induction of ferroptosis-related metabolic disorders by PLGA sustained-release erastin (86.15% ± 2.64% vs. the control group, *P* < 0.05). However, the intensity of its effect was limited by the drug release kinetics (the cumulative release rate in vitro after 48 h was 38.7% ± 2.1%). For the MEPNPs + L group without MF assistance, the passive endocytosis-mediated nanoparticle intake mediated part of the photothermal killing effect (cell viability 69.89% ± 3.40%, *P* < 0.001 vs. the control group). This suggests that the basal level of cell–nanoparticle interactions can generate biological effects under laser irradiation. Notably, magnetic targeting intervention significantly boosted the treatment effect. The MPNPs + MF + L group (without erastin loading) increased the intracellular nanoparticle concentration via the magnetic enrichment effect, and the corresponding photothermal ablation reduced cell viability to 37.93% ± 4.23%. The MEPNPs + MF + L group demonstrated combinational treatment benefits: magnetic navigation promoted accumulation of nanoparticles in tumor cells, and the photothermal effect caused the phase transition of PFP to promote the burst release of drugs (the release rate within 4 min was 82.3% ± 3.6%), ultimately causing a sharp reduction in cell viability to 5.88% ± 1.41% (*P* < 0.001 vs. all other groups). Calcein-AM/propidium iodide double-staining fluorescence imaging quantitatively revealed that the cell death rate in this group reached 93.6% ± 2.8% (Fig. 5A). The FCM annexin V–FITC/propidium iodide detection further confirmed that the proportion of late apoptosis/necrosis was 94.14% ± 2.37% (Fig. 5C and D), forming a tight evidence chain with the viability detection results.

**Fig. 6.**
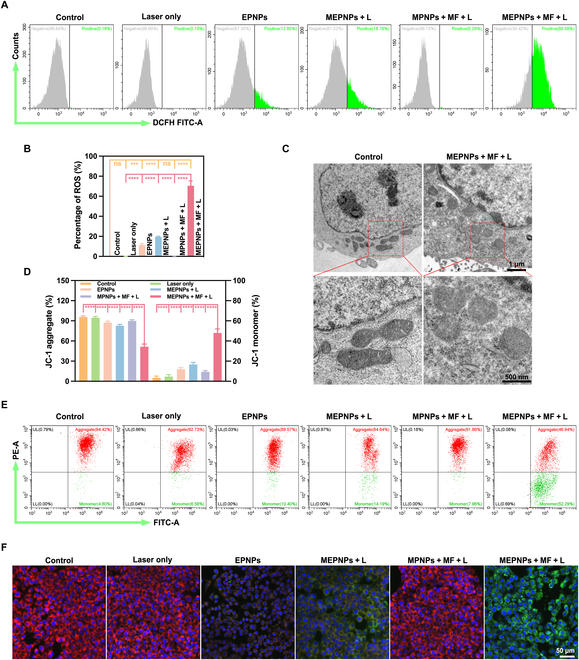
MEPNPs promote oxidative stress and lipid peroxidation in vitro. (A) Flow cytometry (FCM) analysis showing ROS content in 4T1 cells after various treatments. (B) Statistical chart of the total ROS in each group (*n* = 3). (C) TEM showing the morphological changes of tumor mitochondria after various treatments. (D) Statistical chart of the proportion of JC-1 polymers and monomers in the mitochondria of each group of cells. (E) FCM analysis showing the membrane potential. (F) CLSM showing the changes in membrane potential. ***P < 0.001; ****P < 0.0001. DCFH, dichlorodihydrofluorescein; UL, up-left; LL, low-left.

Related biomarkers were systematically detected to analyze the mechanism of ferroptosis in the combinational treatment. The MEPNPs + MF + L group presented typical ferroptosis features; i.e., GSH levels decreased to 32.3% ± 3.4% of the control group (*P* < 0.001), and GSSG/MDA increased to 5.1 ± 0.6 times and 4.7 ± 0.3 times, respectively (Fig. 5E to G). Subsequently, further molecular evidence showed that MEPNPs induce oxidative stress and organelle damage in cells. The oxidative stress characteristics of the MEPNPs + MF + L group were systematically revealed through confocal microscopy imaging and quantitative analysis by FCM. The DCFH-DA probe detection revealed that the ROS level in the treated group was 11.3 ± 0.5 times that of the control group (*P* < 0.001), significantly higher than those of other experimental groups (Fig. 6A and B and Fig. S7A and B). Further monitoring of the lipid peroxidation process using C11-BODIPY probe revealed that the green fluorescence intensity in the MEPNPs + MF + L group was 3.8 ± 0.2 times higher than that in the control group (*P* < 0.001); this indicates that it induces a strong lipid peroxidation cascade reaction (Fig. S8A and B). Mitochondrial dysfunction is the core feature of ferroptosis. JC-1 staining revealed that the mitochondrial membrane potential in the MEPNPs + MF + L group decreased to 53.6% ± 3.1% of that in the control group (*P* < 0.001) (Fig. 6D and E). Moreover, the red/green fluorescence intensity ratio sharply decreased (Fig. 6F). Further, TEM showed that the mitochondria in the treated group presented typical morphological changes of ferroptosis, such as the disappearance of the crista structure, shrinkage of the outer membrane, and decreased mitochondrial matrix density (Fig. 6C). Notably, the number of mitochondria did not show significant changes, and no mitochondria wrapped by autophagosomes were observed, ruling out the possibility of mitochondrial autophagy [42].

**Fig. 7.**
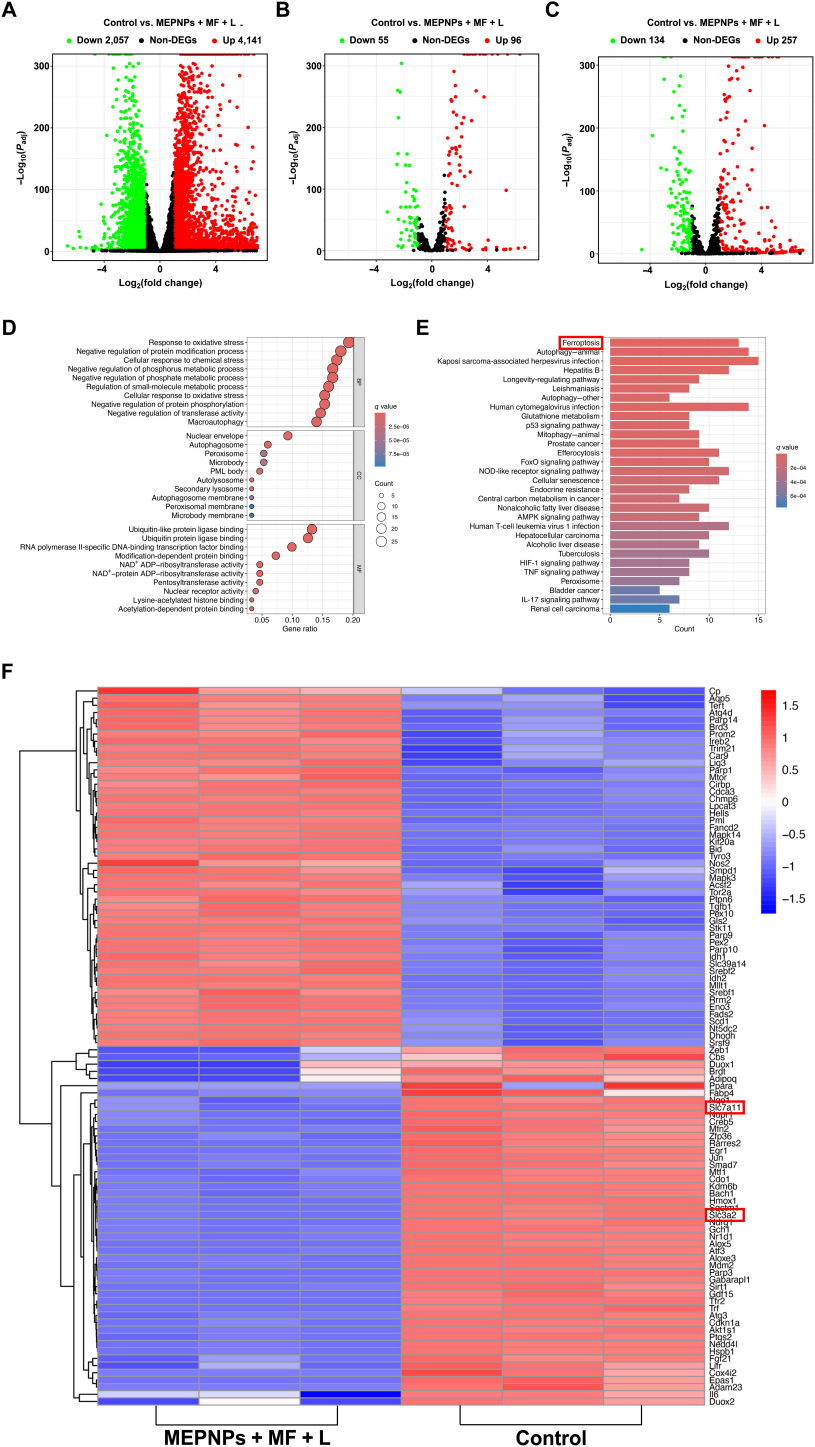
Transcriptome analysis. (A) Volcano plot of differentially expressed genes between the control and MEPNPs + MF + L groups. (B) Volcano plot of differentially expressed ferroptosis-related genes. (C) Volcano plot of differentially expressed ICD-related genes. (D) Gene Ontology (GO) enrichment analysis. (E) Kyoto Encyclopedia of Genes and Genomes (KEGG) enrichment analysis. (F) Heat map showing the differentially expressed genes related to ferroptosis. *n* = 3 biologically independent cell samples per group. DEGs, differentially expressed genes; BP, Biological Process; CC, Cellular Component; MF, Molecular Function.

The global transcriptional reprogramming of 4T1 cells was revealed by RNA sequencing based on the Illumina NovaSeq 6000 platform (Fig. 7A). DESeq2 analysis showed 6,198 differentially expressed genes (|log_2_FC| > 1, false discovery rate < 0.05) in the treatment group; 4,141 genes were significantly up-regulated, whereas 2,057 genes were down-regulated. Through annotation analysis using the FerrDb database, 151 ferroptosis-related genes were significantly regulated (Fig. 7B and F), among which 96 genes had an up-regulation fold of 2.3 to 5.7 (such as SLC39A14, ACSF2, and AQP5, ferroptosis drivers), whereas 55 genes were down-regulated to 18% to 43% of the control group (such as SLC7A11, SLC3A2, and GPX4, antioxidant regulators). GO functional enrichment analysis revealed that the differentially expressed genes were significantly enriched in pathways including “oxidative stress response” (GO:0006979), “peroxisome complex assembly” (GO:0043291), and “NAD(P)H dehydrogenase activity” (GO:0003957) (Fig. 7D). KEGG pathway analysis confirmed that “ferroptosis” (hsa04216) and “glutathione metabolism” (hsa00480) were specifically activated (Fig. 7E); this was highly consistent with the previous lipid peroxidation metabolism detection data. The treatment group also caused significant ICD features. Analysis of 397 ICD-related genes (from the ImmPort database) showed that 257 ICD genes were up-regulated by 1.8 to 4.2 times, and 134 ICD genes were down-regulated to 29% to 65% of the control group (Fig. 7C and Fig. S9). GO analysis revealed that these genes significantly participated in immune activation processes including “antigen-presenting cell activation” (GO:0001773), “interferon-γ response” (GO:0034341), and “T cell proliferation regulation” (GO:0042098) (Fig. S10). KEGG pathway enrichment showed that “Toll-like receptor signaling” (hsa04620), “TNF signaling” (hsa04668), and “T cell differentiation pathway” (hsa05235) were specifically regulated (Fig. S11). More importantly, the results of Western blotting indicated that in the MEPNPs + MF + L group, the expression levels of ICD-related characteristic markers HMGB1 and CRT significantly increased (Fig. S12), which further confirmed that MEPNPs induced the ICD process. This suggests that the treatment may reshape the tumor’s immune microenvironment. Transcriptional data further provided theoretical support for the “cell death–immune activation” dual-effector mechanism produced by MEPNPs in the magnetic–optical acoustic cascade response.

These results collectively elucidate that the magnetic-targeted–photothermal-triggered system yields efficient anti-tumor effects through a triple combinational mechanism, i.e., (a) local high heat mediated by MnFe_2_O_4_, which leads to direct cell ablation; (b) the heat-triggered release of erastin, which inhibits the system Xc^−^ transporter, blocking GSH synthesis; and (c) mitochondrial membrane lipid peroxidation that causes uncoupling of the electron transfer chain, forming a positive feedback loop of “oxidative stress—energy metabolism collapse”. This multitarget attack strategy effectively blocks the adaptive resistance of tumor cells to single-pathway intervention.

### Verification of the spatiotemporal precise temperature control therapy strategy

**Fig. 8.**
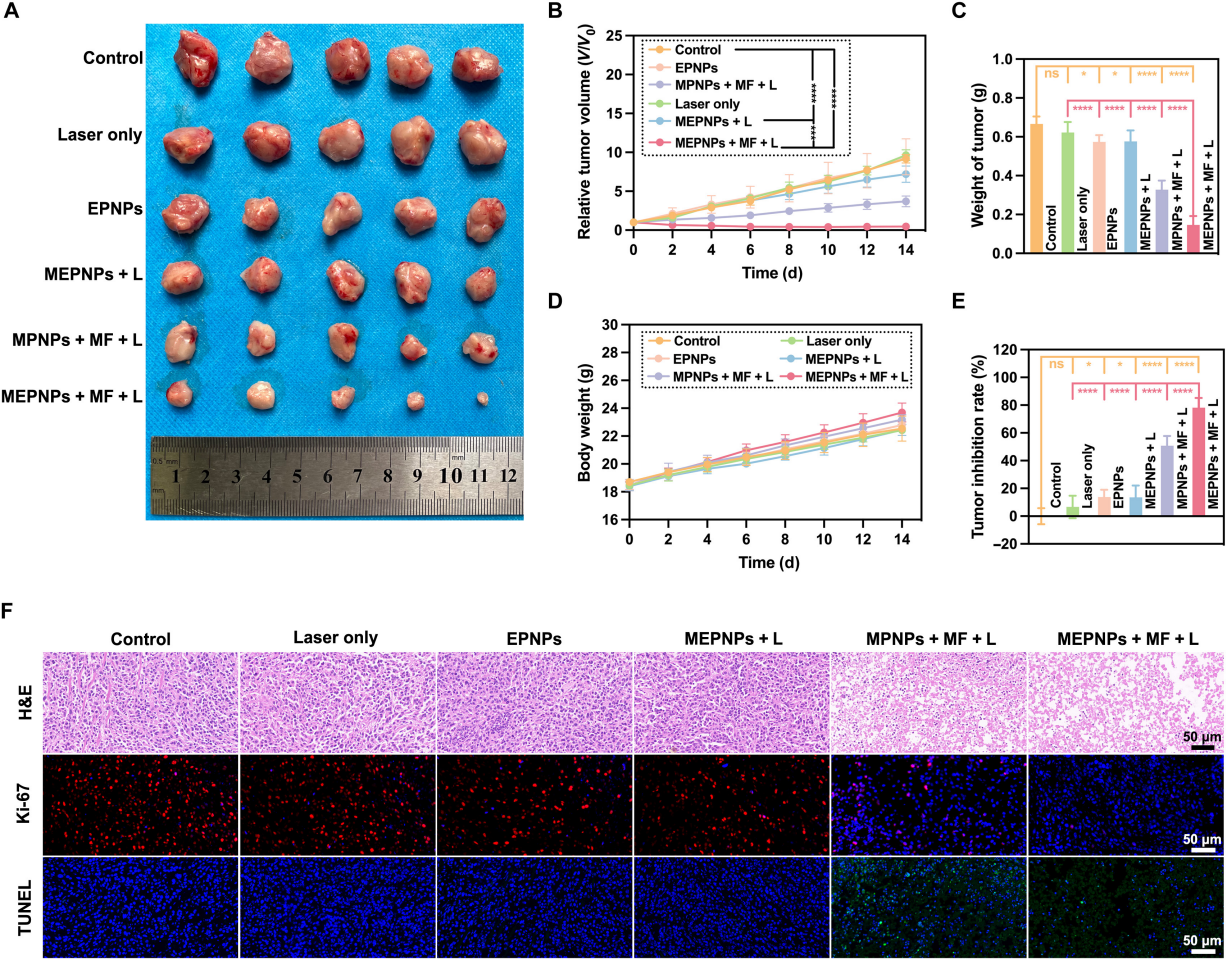
In vivo photothermal performance of the MEPNPs. (A) Images of dissected tumors. (B) Relative tumor volume. (C) Tumor weight. (D) Body weight in various treatment groups. (E) Tumor inhibition rate. (F) Hematoxylin and eosin (H&E), Ki-67 protein, and terminal deoxynucleotidyl transferase dUTP nick end labeling (TUNEL) staining images in tumor tissues after different treatments. *P < 0.05; ****P < 0.0001.

The combined analysis of in vivo fluorescence and multimodal imaging in mice confirmed that the accumulation of MEPNPs in the tumor area reached its peak 4 h following tail vein injection (during the synchronized MF intervention window period). As a result, we formulated a precise treatment timing sequence: NIR laser irradiation (808 nm, 1.0 W/cm^2^) was initiated starting from 4 h following administration. The tumor temperature field distribution was monitored in real time using the FLIR A655sc infrared thermal imager (Fig. S13). Thermodynamic analysis revealed that in the MPNPs + MF + L group (without erastin loading), the photothermal conversion efficiency also increased due to the increase in MnFe_2_O_4_ loading. Ultimately, the local temperature of the tumor increased to 45.2 ± 0.8 °C (Fig. S14A). In contrast, the maximum temperature was kept at 44.8 °C in the MEPNPs + MF + L group under the combinational effect of phase transition energy consumption, which met the mild photothermal treatment category (< 45 °C) at the same time, ensuring effective drug controlled release (Fig. S14B). In vivo anti-tumor efficacy evaluation showed that the MEPNPs + MF + L group demonstrated significant treatment benefits (Fig. S15). After 14 d of treatment, the tumor volume inhibition rate of this group reached 78.08% ± 3.25% (vs. control group, *P* < 0.001) and the tumor weight decreased to 0.21 ± 0.05 g (vs. 1.12 ± 0.15 g of the control group, *P* < 0.001), with no significant weight loss (Fig. 8A to E). Further histopathological analysis further verified the combinational therapeutic mechanism: H&E staining showed that in the MEPNPs + MF + L group, large areas of coagulative necrosis occurred, the Ki-67 positive rate significantly decreased, and TUNEL detection showed an increase in the apoptotic index (Fig. 8F). Despite exhibiting a stronger thermal ablation capacity, the MPNPs + MF + L group showed significantly weaker histopathological changes compared to the MEPNPs + MF + L group, confirming the necessity of ferroptosis combinational enhancement. More importantly, the results of Western blotting showed that COX2 was overexpressed in the MEPNPs + MF + L group, while the expression of GPX4 was significantly down-regulated (Fig. S16). This indicates that MEPNPs also induced the ferroptosis process in vivo.

### Analysis of multi-level immune activation effects

In vitro immune profiling demonstrated that MEPNPs + MF + L treatment significantly enhanced DC maturation. FCM revealed that CD80^+^CD86^+^ double-positive DCs increased to 47.8% ± 1.5%—representing a 3.8-fold elevation versus controls (12.6% ± 1.8%, *P* < 0.001) and significantly exceeding single PTT (MPNPs + MF + L: 38.7% ± 1.8%, *P* < 0.01; Fig. S17A and B). ELISA quantification confirmed the concomitant up-regulation of DC-derived immunostimulatory cytokines: IL-12 (49.5 ± 3.3 pg/ml) and TNF-α (535.9 ± 20.1 pg/ml) increased 3.7-fold and 2.0-fold, respectively (*P* < 0.001; Fig. S17C and D). These findings indicate combinatorial enhancement of antigen presentation by ferroptosis-released tumor antigens and MPTT-induced damage-associated molecular patterns (DAMPs).

**Fig. 9.**
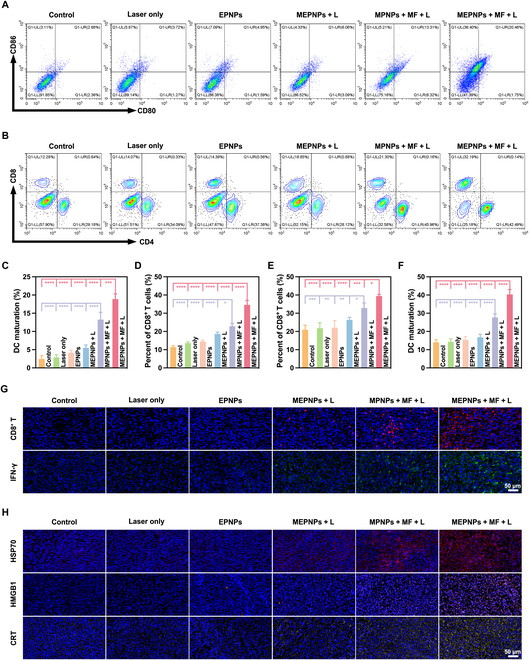
In vivo immune stimulatory effects of MEPNPs. (A) FCM showing DC maturation in primary tumors after different treatments. (B) FCM showing CD8^+^ T cells in the spleens of mice. (C) Statistical chart of DC maturation in primary tumors (*n* = 5). (D) Statistical chart of CD8^+^ T cells in the spleens (*n* = 5). (E) Statistical chart of CD8^+^ T cells in the lymph nodes (*n* = 5). (F) Statistical chart of DC maturation in the spleens (*n* = 5). (G) Immunofluorescence images of CD8^+^ T cells and IFN-γ in tumor tissues. (H) Immunofluorescence images of heat shock protein 70 (HSP70), HMGB1, and CRT in tumor tissues. *P < 0.05; **P < 0.01; ***P < 0.001; ****P < 0.0001.

In vivo dynamic monitoring revealed systemic immune activation. By day 9, tumor-infiltrating DC maturation (CD11c^+^CD80^+^CD86^+^) in the MEPNPs + MF + L group reached 18.87% ± 1.18%—a 7.6-fold increase versus controls (2.47% ± 0.71%, *P* < 0.001; Fig. 9A and C). Significant DC maturation also occurred in lymphoid organs: lymph nodes (40.30% ± 2.21%) and spleen (31.09% ± 1.51%; Fig. 9F and Fig. S18A). Effector T-cell populations expanded substantially, with splenic CD8^+^ T cells increasing to 34.62% ± 2.00% (*P* < 0.001 vs. all groups) and lymph node CD8^+^ T cells to 39.42% ± 0.71% (Fig. 9B, D, and E and Fig. S18B). Immunofluorescence confirmed enhanced tumor CD8^+^ T-cell density and IFN-γ fluorescence intensity (Fig. 9G). Mechanistically, we observed in the treated tumors an increase in calreticulin membrane translocation, extracellular HMGB1, and HSP70 expression (Fig. 9H).

**Fig. 10.**
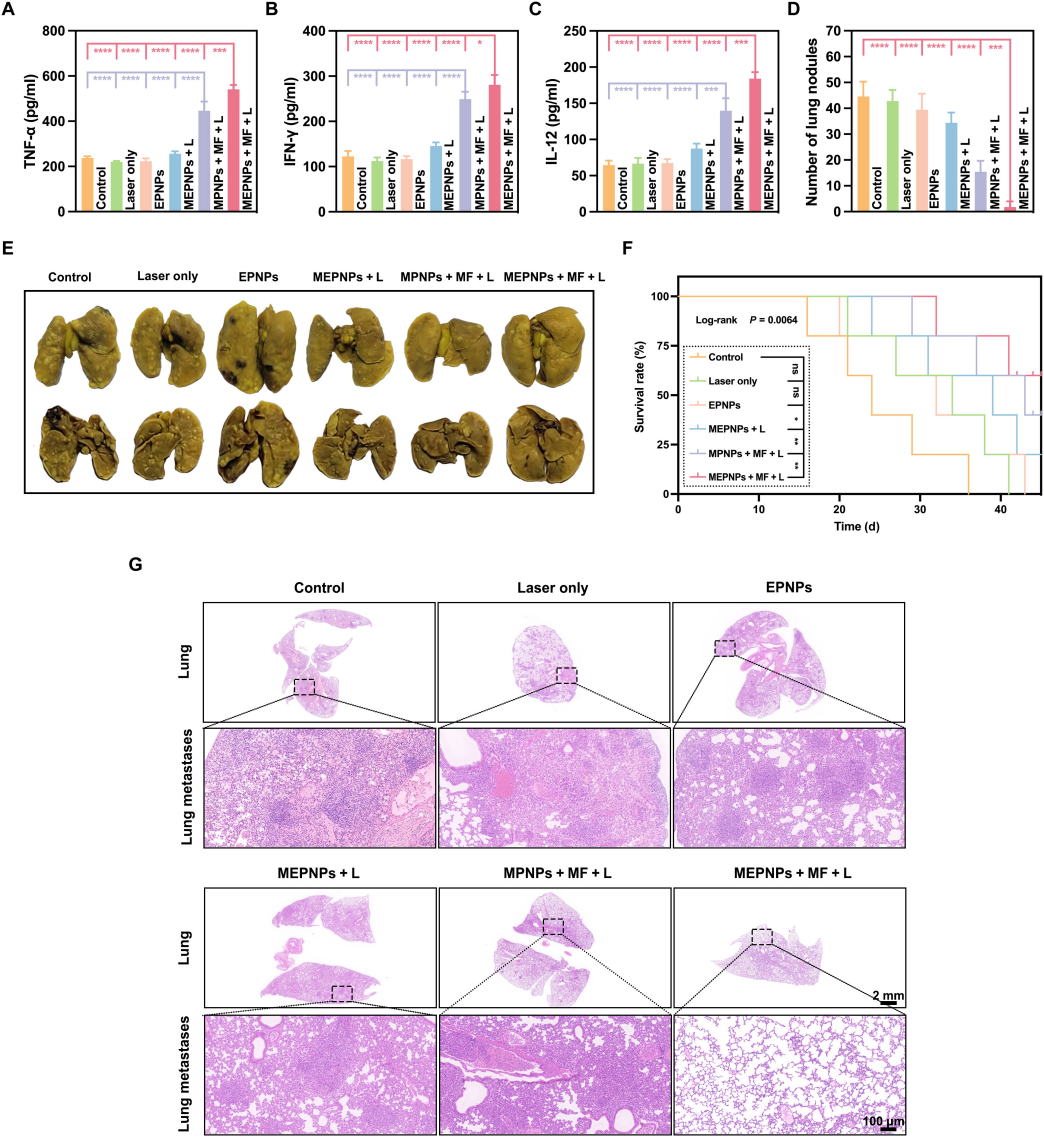
Anti-metastatic effect in vivo. Enzyme-linked immunosorbent assay (ELISA) showing the secretion levels of TNF-α (A), IFN-γ (B), and IL-12 (C) in blood (*n* = 5). (D) Statistical chart of the number of pulmonary metastatic nodules in each treatment group. (E) Images of representative lung metastases in each treatment group. (F) Survival curves of mice in each treatment group. The *P* value of 0.0064 is the log-rank test result of MEPNPs + MF + L versus the control group. (G) H&E staining of resected lung tissue after each treatment. *P < 0.05; **P < 0.01; ***P < 0.001; ****P < 0.0001.

Serological analyses showed elevated cytokines: IL-12 (184.1 ± 7.2 pg/ml), TNF-α (540.9 ± 16.0 pg/ml), and IFN-γ (280.1 ± 17.5 pg/ml) reached 2.3- to 2.9-fold control levels (Fig. 10A to C), indicating tertiary lymphoid structure activation. Crucially, IFN-γ suppresses system Xc^−^ transporter expression (SLC7A11/SLC3A2) [43–45], reducing cystine uptake and GSH synthesis. This indirectly inhibits GPX4 activity, preventing lipid peroxide clearance and establishing a self-enhanced ferroptosis loop. This IFN-γ-mediated amplification of ferroptosis following ICD defines cascade immunogenic ferroptosis [46].

This systemic immune activation conferred significant suppression of distant lung metastases, as evidenced by reduced metastatic nodules and prolonged survival. In the 4T1 lung metastasis model, we found that the number of lung metastatic nodules (1.8 ± 0.2 vs. control group 44.6 ± 5.7, *P* < 0.001) and the metastatic load (H&E metastasis area percentage) in the MEPNPs + MF + L group were significantly reduced (Fig. 10D, E, and G). Compared with the control group, the survival period of the MEPNPs + MF + L group was significantly prolonged (*P* = 0.0064, log-rank test), with a median survival period exceeding 45 d, while that of the control group was 24 d (Fig. 10F).

### The spatiotemporal contributions of key components to therapeutic efficacy

The outstanding tumor suppression effect (78.08% ± 3.25%) and significant anti-metastasis efficacy of the MEPNPs + MF + L group are attributed to the synergistic effects of 4 core elements: (a) Magnetic targeting (MF) acts as a spatial gatekeeper, increasing the accumulation of nanoparticles at the tumor site by 6.1 times (Fig. 3F). Without MF, relying solely on passive EPR-mediated delivery (MEPNPs + L), the tumor suppression rate was only 42.7% ± 4.1%, highlighting the indispensability of MF in overcoming biological barriers (Fig. 8E). (b) NIR irradiation (L) serves as a spatiotemporal trigger and plays a crucial role: on the one hand, laser-induced PFP phase transition generates microbubbles within 4 min, enhancing US contrast by over 27 times (Fig. 4I). This acoustic signal provides real-time feedback on drug release within the tumor during treatment. On the other hand, the local high temperature (44.8 ± 0.8 °C, Fig. S14B) generated after laser irradiation not only directly damages tumor cells but also sensitizes them to ferroptosis by liquefying the cell membrane and enhancing erastin diffusion. Although the EPNPs group without laser showed a slow erastin release (38.7% ± 2.1% at 48 h, Fig. S2J), the PFP phase transition in laser-triggered MEPNPs accelerated the release to 82.3% ± 3.6% within 4 min. This mechanical disruption of the PLGA shell enables immediate erastin administration, bypassing the kinetic limitations of passive diffusion. (c) Erastin loading is the molecular effector of the ferroptosis–immune cross talk: in MPNPs + MF + L (without erastin), only photothermal ablation achieved moderate cytotoxicity (cell survival rate: 37.93% ± 4.23%, Fig. 5B) but failed to induce strong ICD or systemic immunity (Fig. 9C and Fig. S17). However, in MEPNPs + MF + L (with erastin), erastin reduced the tumor GSH level to 32.3% ± 3.4% of the control group (Fig. 5E), down-regulated GPX4 (Fig. S16), and triggered a lipid peroxidation cascade (MDA increased 4.7 times, Fig. 5G). Crucially, compared to the MPNPs + MF + L (without erastin) group, MEPNPs + MF + L (erastin-loaded group) showed further increased expression of ICD markers (HMGB1/CRT, Fig. S12), enhanced CD8^+^ T-cell infiltration (Fig. 9C), and increased IFN-γ secretion (Fig. 10B). (d) Synergistic effects exceed additivity: the combinatorial group (MEPNPs + MF + L) reduced tumor survival rate to 5.88% ± 1.41% in vitro (Fig. 5B), significantly outperforming subgroups lacking MF (69.89% ± 3.40%), erastin (37.93% ± 4.23%), or laser (86.15% ± 2.64%). Moreover, in vivo, it outperformed subgroups lacking MF, erastin, or laser in terms of tumor suppression rate (Fig. 8), systemic immune activation (Fig. 9), and distant metastasis inhibition rate (Fig. 10). This confirms that magnetic targeting achieved sufficient drug deposition within the tumor, and the NIR-controlled PFP phase transition ensured the precise release of erastin. Erastin-driven ferroptosis further transformed the “cold” tumor into an immunogenic “hot” phenotype, a trio that no single therapy could achieve.

## Conclusion

In summary, we have engineered a magneto-photo-acoustic theranostic platform (MEPNPs) that integrates trimodal imaging guidance with spatiotemporally controlled therapeutic modalities, demonstrating transformative potential for TNBC management. The 3-step cascade reaction enhanced the therapeutic effect: (a) Magnetic targeting: Superparamagnetic MnFe_2_O_4_ can achieve enhanced accumulation at the tumor site via external magnetic navigation, delivering therapeutic doses precisely to tumor margins. (b) Photothermal-phase transition synergy: NIR-triggered photothermal conversion couples with PFP phase transition, enabling simultaneous localized hyperthermia and pulsatile erastin release. This dual action depletes GPX4 while elevating lipid peroxides, initiating a self-amplifying ferroptotic loop. (c) Immunogenic conversion: DAMP/HSP70 release transforms “cold” tumors into immunologically “hot” phenotypes, leading to increased CD8^+^ T-cell infiltration and DC maturation, ultimately establishing systemic anti-tumor immunity against metastases. While challenges in scalable manufacturing and chronic biosafety require further investigation, this work establishes a paradigm-shifting strategy that bridges nanomaterial physics with tumor immunometabolism. The MEPNP platform not only overcomes TNBC’s therapeutic resistance through spatially confined multimechanism attack but also provides a blueprint for developing next-generation theranostic systems against refractory solid malignancies.

## Ethical Approval

All animal experiments were in accordance with the Guide for Care and Use of Laboratory Animals and approved by the Animal Ethics Committee of Chongqing Medical University.

## Data Availability

The data that support the findings of this study are available on request from the corresponding authors.

## References

[1] Yu L, Liebenberg K, Shen Y, Liu F, Xu Z, Hao X, Wu L, Zhang W, Chan HL, Wei B, et al. Tumor-derived arachidonic acid reprograms neutrophils to promote immune suppression and therapy resistance in triple-negative breast cancer. Immunity. 2025;58(4):909–925.e7. 40157359 10.1016/j.immuni.2025.03.002 PMC11981829

[2] Yang F, Xiao Y, Ding JH, Jin X, Ma D, Li DQ, Shi JX, Huang W, Wang YP, Jiang YZ, et al. Ferroptosis heterogeneity in triple-negative breast cancer reveals an innovative immunotherapy combination strategy. Cell Metab. 2023;35(1):84–100.e8. 36257316 10.1016/j.cmet.2022.09.021

[3] Sui XY, Cao SW, Song XQ, Liu XY, Chen C, Yan Q, Wang ZQ, Zhang WJ, Ma LX, Jin X, et al. Correction: MORF4L2 induces immunosuppressive microenvironment and immunotherapy resistance through GRHL2/MORF4L2/H4K12Ac/CSF1 axis in triple-negative breast cancer. Biomark Res. 2025;13(1):29. 39948640 10.1186/s40364-025-00743-9 PMC11827472

[4] Mokhtarpour K, Razi S, Rezaei N. Correction: Ferroptosis as a promising targeted therapy for triple negative breast cancer. Breast Cancer Res Treat. 2024;207(3):515. 39078443 10.1007/s10549-024-07438-z

[5] Mokhtarpour K, Razi S, Rezaei N. Ferroptosis as a promising targeted therapy for triple negative breast cancer. Breast Cancer Res Treat. 2024;207(3):497–513. 38874688 10.1007/s10549-024-07387-7

[6] Herrera-Abreu MT, Guan J, Khalid U, Ning J, Costa MR, Chan J, Li Q, Fortin JP, Wong WR, Perampalam P, et al. Inhibition of GPX4 enhances CDK4/6 inhibitor and endocrine therapy activity in breast cancer. Nat Commun. 2024;15(1):9550. 39500869 10.1038/s41467-024-53837-7 PMC11538343

[7] Li K, Lin C, Li M, Xu K, He Y, Mao Y, Lu L, Geng W, Li X, Luo Z, et al. Multienzyme-like reactivity cooperatively impairs glutathione peroxidase 4 and ferroptosis suppressor protein 1 pathways in triple-negative breast cancer for sensitized ferroptosis therapy. ACS Nano. 2022;16(2):2381–2398. 35041395 10.1021/acsnano.1c08664

[8] Li Y, Liu J, Chen Y, Weichselbaum RR, Lin W. Nanoparticles synergize ferroptosis and cuproptosis to potentiate cancer immunotherapy. Adv Sci. 2024;11(23): Article e2310309. 10.1002/advs.202310309 PMC11187894 38477411

[9] Liu B, Duan H, Sun L, Liu Z, Sun Z, Chu H. Core–shell structured metal–organic frameworks for pH-triggered combination photodynamic/chemotherapy-based cancer treatment. Biomater Res. 2025;29:0138. 39844866 10.34133/bmr.0138 PMC11751201

[10] Tian H, Li W, Wang G, Tian Y, Yan J, Yu X, Yan Z, Feng Y, Dai Y. Metal-phenolic nanomaterial with organelle-level precision primes antitumor immunity via mtDNA-dependent cGAS-STING activation. Angew Chem Int Ed Engl. 2024;63(50): Article e202411498. 39143745 10.1002/anie.202411498

[11] Li J, Wu Y, Li Y, Zhu H, Zhang Z, Li Y. Glutathione-disrupting nanotherapeutics potentiate ferroptosis for treating luminal androgen receptor-positive triple-negative breast cancer. ACS Nano. 2024;18(39):26585–26599. 39287044 10.1021/acsnano.4c04322

[12] Wang R, Xiao Y, Zhang Z, Huang X, Zhu W, Ma X, Feng F, Liu W, Han L, Qu W. Simplified gambogic acid prodrug nanoparticles to improve efficiency and reduce toxicity for clinical translation potential. Adv Healthc Mater. 2024;13(31): Article e2401950. 39276002 10.1002/adhm.202401950

[13] Zhang H, Li L, Liu XL, Jiao J, Ng CT, Yi JB, Luo YE, Bay BH, Zhao LY, Peng ML, et al. Ultrasmall ferrite nanoparticles synthesized *via* dynamic simultaneous thermal decomposition for high-performance and multifunctional *t*_1_ magnetic resonance imaging contrast agent. ACS Nano. 2017;11(4):3614–3631. 28371584 10.1021/acsnano.6b07684

[14] Amiri M, Salavati-Niasari M, Akbari A. Magnetic nanocarriers: Evolution of spinel ferrites for medical applications. Adv Colloid Interf Sci. 2019;265:29–44. 10.1016/j.cis.2019.01.003 30711796

[15] Yang K, Yu G, Tian R, Zhou Z, Deng H, Li L, Yang Z, Zhang G, Liu D, Wei J, et al. Oxygen-evolving manganese ferrite nanovesicles for hypoxia-responsive drug delivery and enhanced cancer chemoimmunotherapy. Adv Funct Mater. 2021;31(11):2008078.

[16] Miao Y, Xie Q, Zhang H, Cai J, Liu X, Jiao J, Hu S, Ghosal A, Yang Y, Fan H. Composition-tunable ultrasmall manganese ferrite nanoparticles: Insights into their *in vivo* T_1_ contrast efficacy. Theranostics. 2019;9(6):1764–1776. 31037137 10.7150/thno.31233 PMC6485191

[17] Yao J, Yang Z, Huang L, Yang C, Wang J, Cao Y, Hao L, Zhang L, Zhang J, Li P, et al. Low-intensity focused ultrasound-responsive ferrite-encapsulated nanoparticles for atherosclerotic plaque neovascularization theranostics. Adv Sci. 2021;8(19): Article e2100850. 10.1002/advs.202100850 PMC8498883 34382370

[18] Yang Z, Yao J, Wang J, Zhang C, Cao Y, Hao L, Yang C, Wu C, Zhang J, Wang Z, et al. Ferrite-encapsulated nanoparticles with stable photothermal performance for multimodal imaging-guided atherosclerotic plaque neovascularization therapy. Biomater Sci. 2021;9:5652–5664. 34259244 10.1039/d1bm00343g

[19] Liu M, Lu Y, Zhao J, Yin Y, Cao J, Wu L, Shen S. Artemisinin and salinomycin co-loaded nanozymes to boost cascade ROS accumulation for augmented tumor ferroptosis. Colloids Surf B Biointerfaces. 2025;245: Article 114352. 39500100 10.1016/j.colsurfb.2024.114352

[20] Zhang G, Li N, Qi Y, Zhao Q, Zhan J, Yu D. Synergistic ferroptosis-gemcitabine chemotherapy of the gemcitabine loaded carbonaceous nanozymes to enhance the treatment and magnetic resonance imaging monitoring of pancreatic cancer. Acta Biomater. 2022;142:284–297. 35151925 10.1016/j.actbio.2022.02.006

[21] Li Y, Zhao X, Liu X, Cheng K, Han X, Zhang Y, Min H, Liu G, Xu J, Shi J, et al. A bioinspired nanoprobe with multilevel responsive *T*_1_-weighted MR signal-amplification illuminates ultrasmall metastases. Adv Mater. 2020;32(4):1906799. 10.1002/adma.201906799 31799765

[22] Sun Z, Shao C, Hao S, Zhang J, Ren W, Wang B, Xiao L, Lei H, Liu TX, Yuan Z, et al. Lignin-based photothermal materials: Bridging sustainability and high-efficiency energy conversion. Adv Sci. 2025;12(20): Article e2501259. 10.1002/advs.202501259 PMC12120746 40279516

[23] Kim G, Luo Y, Shin M, Bouffard J, Bae J, Kim Y. Making the brightest ones dim: Maximizing the photothermal conversion efficiency of BODIPY-based photothermal agents. Adv Healthc Mater. 2024;13(19): Article e2400885. 38573765 10.1002/adhm.202400885

[24] Xie X, Dong Y, Zhang Y, Xie Z, Peng X, Huang Y, Yang W, Li B, Zhang Q. Readily constructed squaraine J-aggregates with an 86.0 % photothermal conversion efficiency for photothermal therapy. Bioact Mater. 2025;43:460–470. 40115878 10.1016/j.bioactmat.2024.09.031 PMC11923431

[25] McGinnis CS, Miao Z, Superville D, Yao W, Goga A, Reticker-Flynn NE, Winkler J, Satpathy AT. The temporal progression of lung immune remodeling during breast cancer metastasis. Cancer Cell. 2024;42(6):1018–1031.e6. 38821060 10.1016/j.ccell.2024.05.004 PMC11255555

[26] Chen Q, Zhang L, Li L, Tan M, Liu W, Liu S, Xie Z, Zhang W, Wang Z, Cao Y, et al. Cancer cell membrane-coated nanoparticles for bimodal imaging-guided photothermal therapy and docetaxel-enhanced immunotherapy against cancer. J Nanobiotechnol. 2021;19(1):449. 10.1186/s12951-021-01202-x PMC8710014 34952587

[27] Bort G, Lux F, Dufort S, Crémillieux Y, Verry C, Tillement O. EPR-mediated tumor targeting using ultrasmall-hybrid nanoparticles: From animal to human with theranostic AGuIX nanoparticles. Theranostics. 2020;10:1319–1331. 31938067 10.7150/thno.37543 PMC6956799

[28] Kang H, Rho S, Stiles WR, Hu S, Baek Y, Hwang DW, Kashiwagi S, Kim MS, Choi HS. Size-dependent EPR effect of polymeric nanoparticles on tumor targeting. Adv Healthc Mater. 2020;9(1): Article e1901223. 31794153 10.1002/adhm.201901223 PMC7224408

[29] Cullion K, Ostertag-Hill CA, Tang W, Pan M, Kohane DS. Size-dependent nanoparticle accumulation in venous malformations. J Vasc Anom. 2024;5(4): Article e00103. 10.1097/JOVA.0000000000000103 PMC11670902 39734473

[30] Blanco E, Shen H, Ferrari M. Principles of nanoparticle design for overcoming biological barriers to drug delivery. Nat Biotechnol. 2015;33(9):941–951. 26348965 10.1038/nbt.3330 PMC4978509

[31] He Y, Lei L, Cao J, Yang X, Cai S, Tong F, Huang D, Mei H, Luo K, Gao H, et al. A combinational chemo-immune therapy using an enzyme-sensitive nanoplatform for dual-drug delivery to specific sites by cascade targeting. Sci Adv. 2021;7(6):eaba0776. 33547067 10.1126/sciadv.aba0776 PMC7864565

[32] Liu W, Xiang H, Tan M, Chen Q, Jiang Q, Yang L, Cao Y, Wang Z, Ran H, Chen Y. Nanomedicine enables drug-potency activation with tumor sensitivity and hyperthermia synergy in the second near-infrared biowindow. ACS Nano. 2021;15(4):6457–6470. 33750100 10.1021/acsnano.0c08848

[33] Zeng J, Goldfeld D, Xia Y. A plasmon-assisted optofluidic (PAOF) system for measuring the photothermal conversion efficiencies of gold nanostructures and controlling an electrical switch. Angew Chem Int Ed Engl. 2013;52(15):4169–4173. 23494970 10.1002/anie.201210359 PMC3757564

[34] Hessel CM, Pattani VP, Rasch M, Panthani MG, Koo B, Tunnell JW, Korgel BA. Copper selenide nanocrystals for photothermal therapy. Nano Lett. 2011;11(6):2560–2566. 21553924 10.1021/nl201400z PMC3111000

[35] Li Z, Lu J, Ji T, Xue Y, Zhao L, Zhao K, Jia B, Wang B, Wang J, Zhang S, et al. Self-healing hydrogel bioelectronics. Adv Mater. 2024;36(21): Article e2306350. 37987498 10.1002/adma.202306350

[36] Wang Y, Li H, Lin J, Li Y, Zhang K, Li H, Fu Q, Jiang Y. Engineering nanozyme immunomodulator with magnetic targeting effect for cascade-enzyodynamic and ultrasound-reinforced metallo-immunotherapy in prostate carcinoma. Nat Commun. 2025;16(1):1876. 39987131 10.1038/s41467-025-57190-1 PMC11846840

[37] Chen J, Ren T, Xie L, Hu H, Li X, Maitusong M, Zhou X, Hu W, Xu D, Qian Y, et al. Enhancing aortic valve drug delivery with PAR2-targeting magnetic nano-cargoes for calcification alleviation. Nat Commun. 2024;15(1):557. 38228638 10.1038/s41467-024-44726-0 PMC10792006

[38] Wang G, Jiang Y, Xu J, Shen J, Lin T, Chen J, Fei W, Qin Y, Zhou Z, Shen Y, et al. Unraveling the plasma protein corona by ultrasonic cavitation augments active-transporting of liposome in solid tumor. Adv Mater. 2023;35(9): Article e2207271. 36479742 10.1002/adma.202207271

[39] Wang G, Zhang C, Jiang Y, Song Y, Chen J, Sun Y, Li Q, Zhou Z, Shen Y, Huang P. Ultrasonic cavitation-assisted and acid-activated transcytosis of liposomes for universal active tumor penetration. Adv Funct Mater. 2021;31(34):2102786.

[40] Chowdhury SM, Abou-Elkacem L, Lee T, Dahl J, Lutz AM. Ultrasound and microbubble mediated therapeutic delivery: Underlying mechanisms and future outlook. J Control Release. 2020;326:75–90. 32554041 10.1016/j.jconrel.2020.06.008

[41] Rix A, Piepenbrock M, Flege B, von Stillfried S, Koczera P, Opacic T, Simons N, Boor P, Thoroe-Boveleth S, Deckers R, et al. Effects of contrast-enhanced ultrasound treatment on neoadjuvant chemotherapy in breast cancer. Theranostics. 2021;11:9557–9570. 34646386 10.7150/thno.64767 PMC8490514

[42] Sun Q, Qian Y, Wang J, Wang M, Zhao G, Wang G, Li C. Lanthanide nanocrystals-based direct triple-state energy converters for mitophagy and ferroptosis-induced antitumor immunoreaction. J Control Release. 2025;384: Article 113887. 40436350 10.1016/j.jconrel.2025.113887

[43] Wang J, Zhang W, Xie Z, Wang X, Sun J, Ran F, Jiang W, Liu Y, Wang Z, Ran H, et al. NIR-responsive copper nanoliposome composites for cascaded ferrotherapy via ferroptosis actived ICD and IFN-γ released. Biomaterials. 2024;308: Article 122570. 38636133 10.1016/j.biomaterials.2024.122570

[44] Zhao G, Wang X, Lei H, Ruan N, Yuan B, Tang S, Ni N, Zuo Z, Xun L, Luo M, et al. Serum HMGB-1 released by ferroptosis and necroptosis as a novel potential biomarker for systemic lupus erythematosus. Int Immunopharmacol. 2024;140: Article 112886. 39128419 10.1016/j.intimp.2024.112886

[45] Chen C, Wang Z, Jia S, Zhang Y, Ji S, Zhao Z, Kwok RTK, Lam JWY, Ding D, Shi Y, et al. Evoking highly immunogenic ferroptosis aided by intramolecular motion-induced photo-hyperthermia for cancer therapy. Adv Sci. 2022;9(10): Article e2104885. 10.1002/advs.202104885 PMC8981454 35132824

[46] Wang H, Jiao D, Feng D, Liu Q, Huang Y, Hou J, Ding D, Zhang W. Transformable supramolecular self-assembled peptides for cascade self-enhanced ferroptosis primed cancer immunotherapy. Adv Mater. 2024;36(21): Article e2311733. 38339920 10.1002/adma.202311733

