# Supplementary material for: Magneto-photo-acoustic Nanotheranostics Orchestrate Ferroptosis–Immune Cross Talk for Spatiotemporally Amplified Triple-Negative Breast Cancer Therapy
Source: Biomater Res. 2025 Oct 9;29:0258. doi: 10.34133/bmr.0258 (PMC12509091; doi:10.34133/bmr.0258)
Supplement: Supplementary 1 — Figs. S1 to S18 [file bmr.0258.f1.docx]

Supplementary Materials

Magneto-Photo-Acoustic Nanotheranostics Orchestrate Ferroptosis-Immune Crosstalk for Spatiotemporally Amplified Triple-Negative Breast Cancer Therapy

*Yibo Qiu^1, 2^, Huan Wu^4^, Zijing Lin^5^, Jieqi Chen^6^, Shiqi Tian^7^, Zhigang Wang^2, 3^, Haitao Ran^2, 3^, Yingxiong Wang^1, *^, Long Cheng^2, 3, *^*

1 School of Basic Medical Sciences, Chongqing Medical University, Chongqing 400016, People's Republic of China

2 Chongqing Key Laboratory of Ultrasound Molecular Imaging and Therapy, the Second Affiliated Hospital of Chongqing Medical University, Chongqing 400010, People's Republic of China

3 Department of Ultrasound, the Second Affiliated Hospital of Chongqing Medical University, Chongqing 400010, People's Republic of China

4 Department of Obstetrics and Gynecology, the Second Affiliated Hospital of Chongqing Medical University, Chongqing 400010, People's Republic of China

5 Department of Breast and Thyroid Surgery, the Second Affiliated Hospital of Chongqing Medical University, Chongqing 400010, People's Republic of China

6 Department of Breast, Thyroid and Hernia Surgery, Yongchuan District People's Hospital, Chongqing City, Chongqing 402160, People's Republic of China

^7^Department of General Medicine,the Affiliate Yongchuan Hospital Of Chongqing Medical University,Chongqing 402160, People's Republic of China.

* Corresponding Author: Yingxiong Wang, Ph.D., School of Basic Medical Sciences, Chongqing Medical University, Chongqing 400016, People's Republic of China. E-mail address: [1600347366@qq.com](mailto:1600347366@qq.com). Long Cheng, M.D., Department of Ultrasound, the Second Affiliated Hospital of Chongqing Medical University, Chongqing 400010, People's Republic of China. E-mail address: [304188@hospital.cqmu.edu.cn](mailto:302118@cqmu.edu.cn).

**
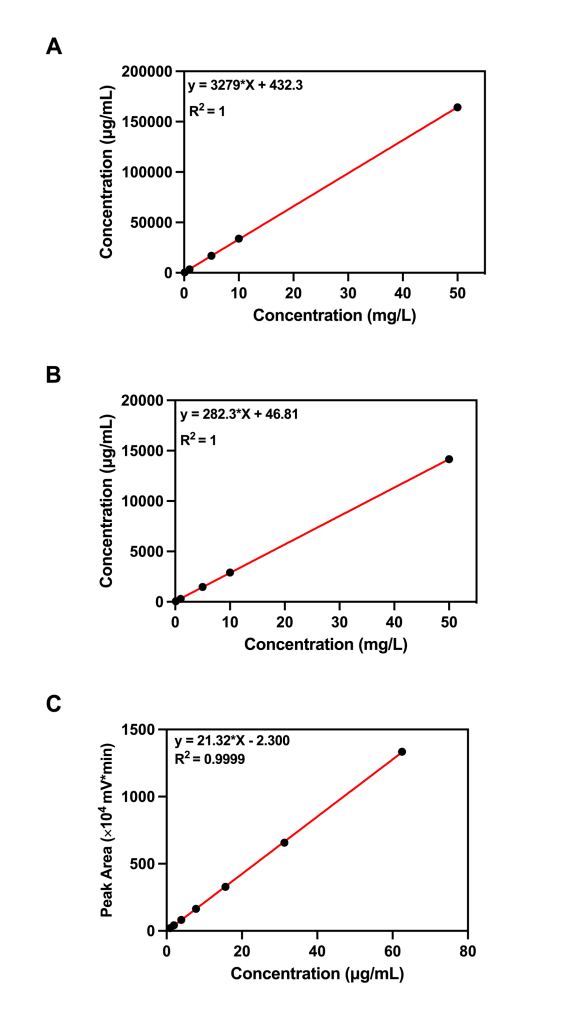
**

**Fig. S1.** **The standard curve of Mn (A), Fe (B), and free Erastin.**


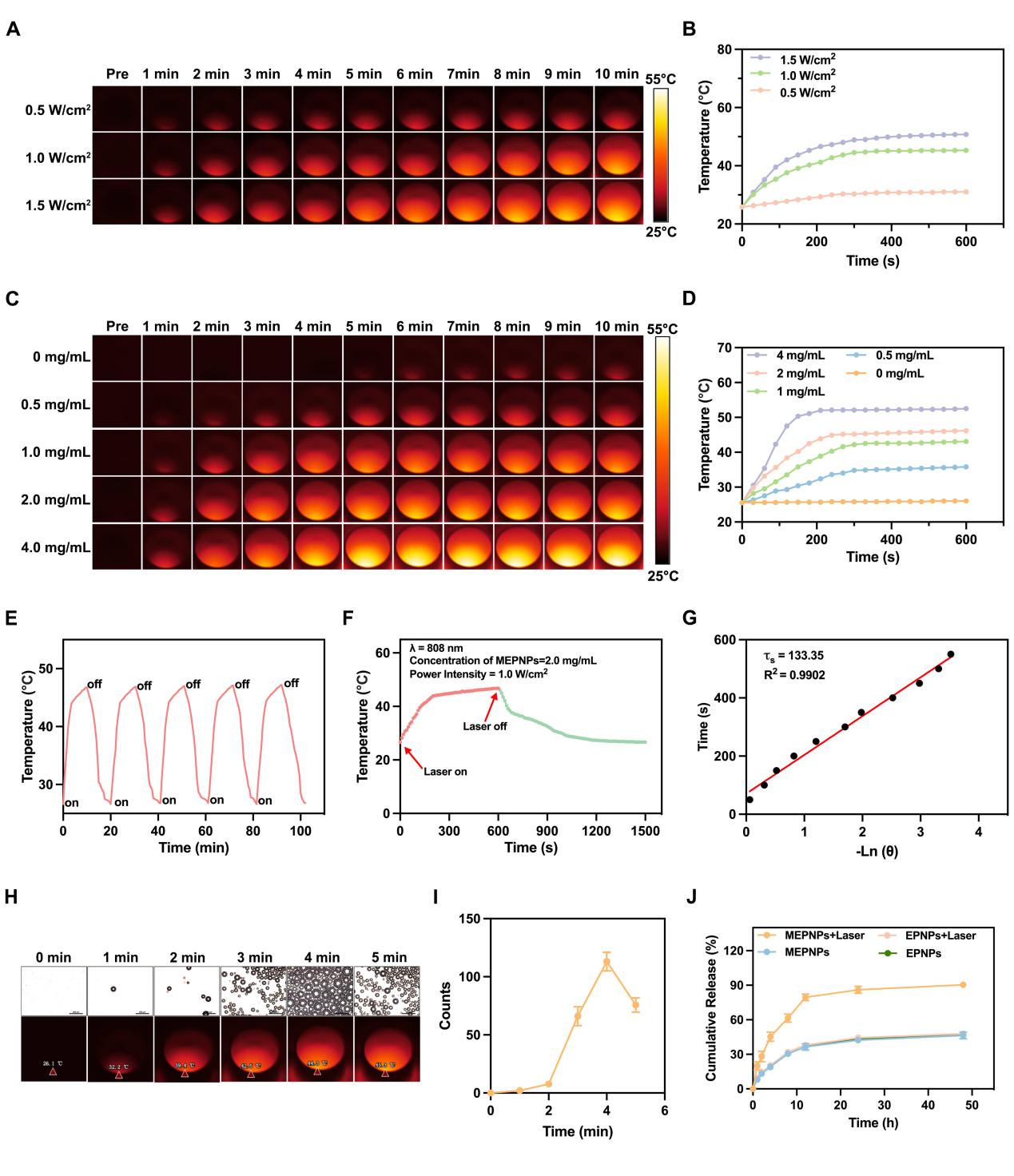


**Fig. S2. Photothermal conversion and photo-controlled drug release. (A, B) Temperature elevation curves of MEPNPs with various laser powers. (C, D) Temperature elevation curves of MEPNPs at various concentrations. (E) Temperature elevation curves of MEPNPs with laser on/off. (F) Photo-to-heat conversion capability of MEPNPs aqueous dispersion upon 808 nm laser exposure (1.0 W/cm2). (G) The time constant was calculated from the cooling period. (H) Microbubble pattern under a light microscope after laser irradiation at different times. (I) The number of microbubbles after laser irradiation at different times. (J) Erastin release from MEPNPs/ EPNPs with or without laser irradiation.**

**
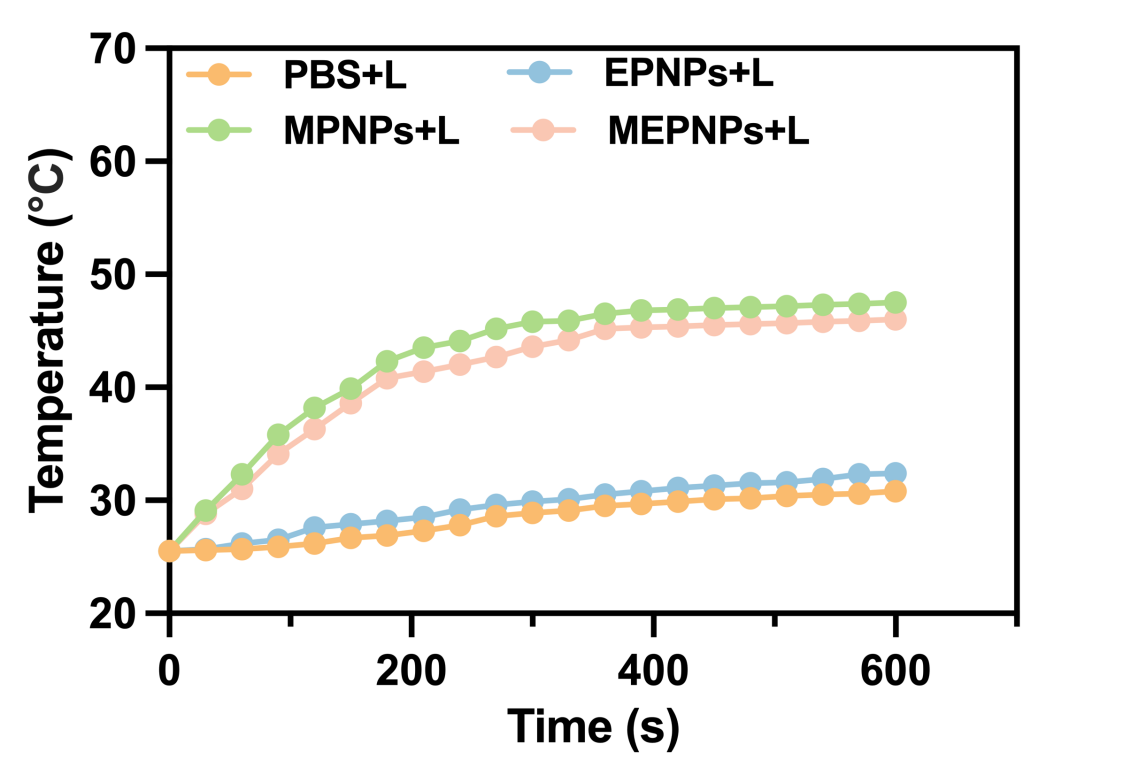
**

**Fig. S3. Temperature change curves of different solutions during laser irradiation.**

**
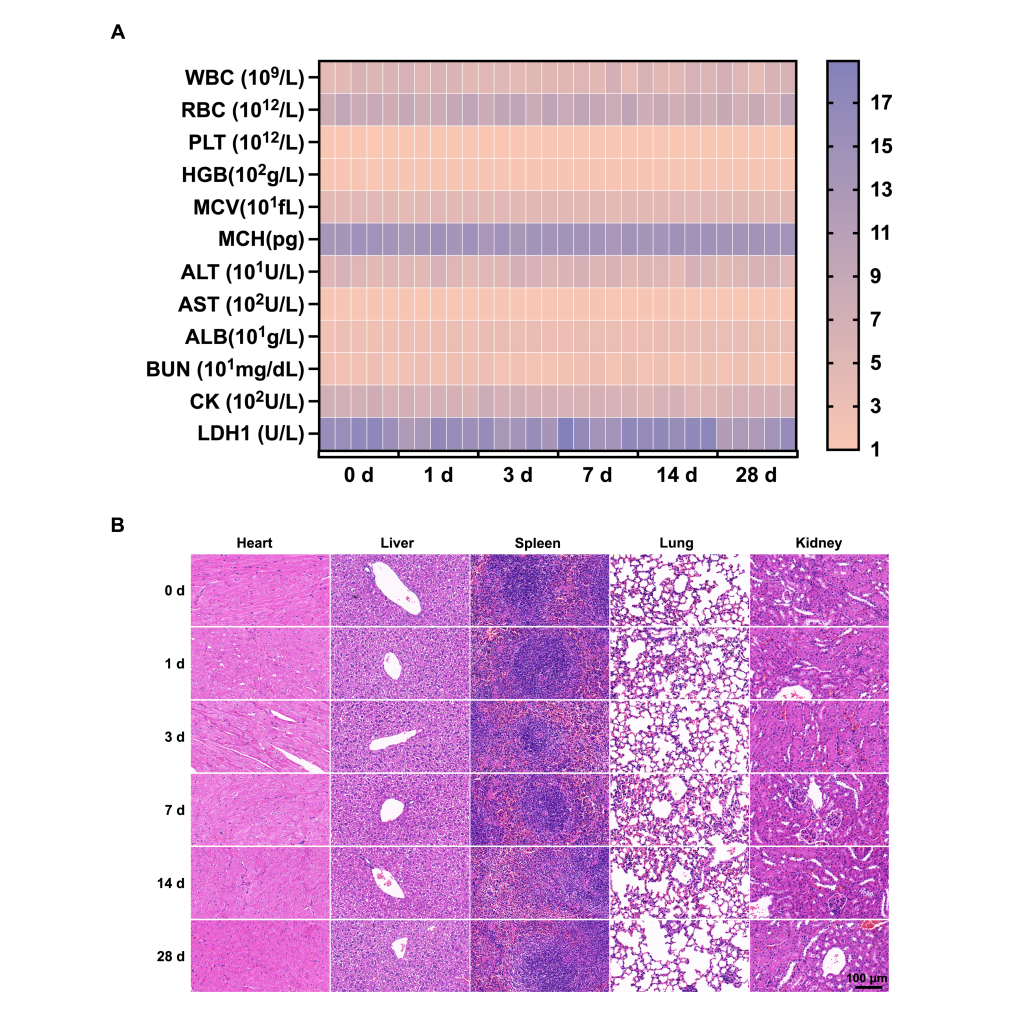
**

**Fig. S4.** **(A) Routine blood and blood biochemistry analysis in mice sacrificed at different time intervals after intravenous administration of MEPNPs. (B) H&E staining of the major organs in mice sacrificed at different time intervals after intravenous injection of MEPNPs.**


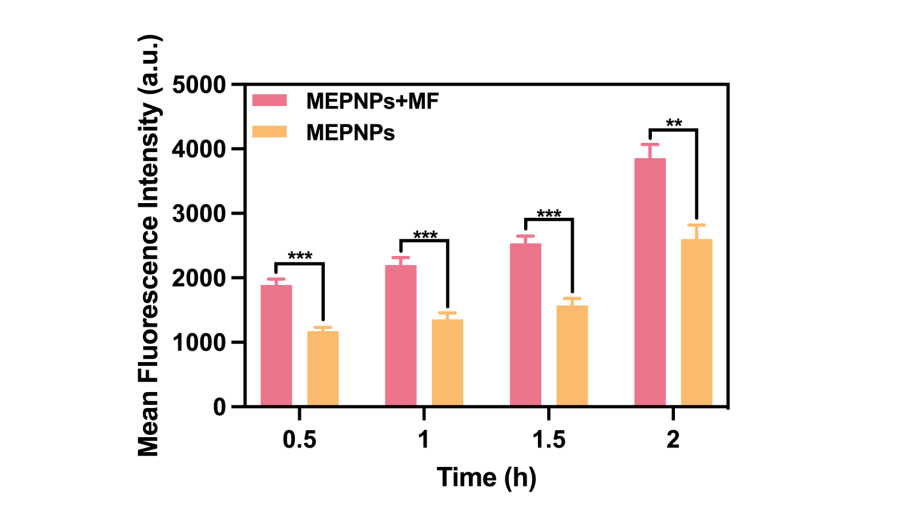


**Fig. S5. The mean fluorescence intensity of MEPNPs uptake by cells in the presence or absence of magnetic field.**

**
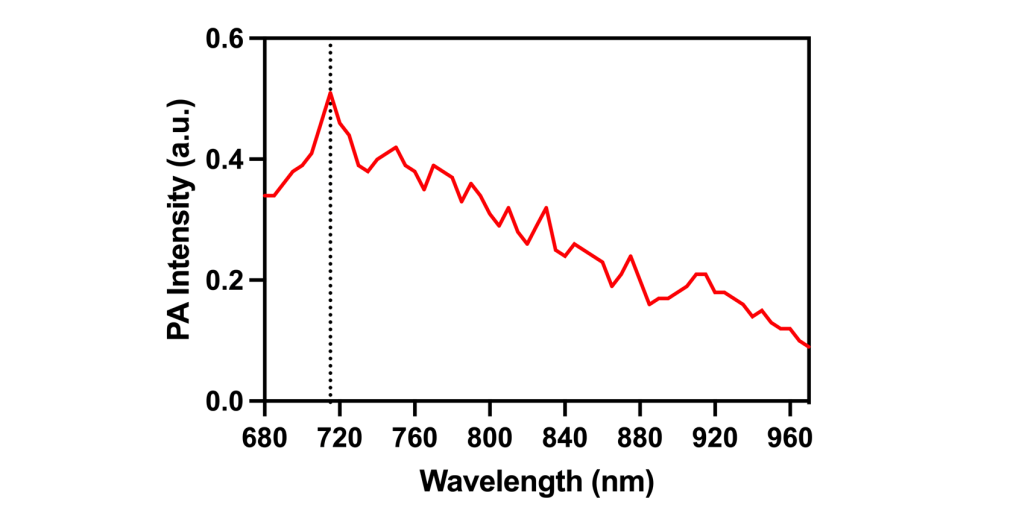
**

**Fig. S6. Photoacoustic intensity of the MEPNPs under full-spectrum scanning (ranging from 680 to 960 nm).**

**
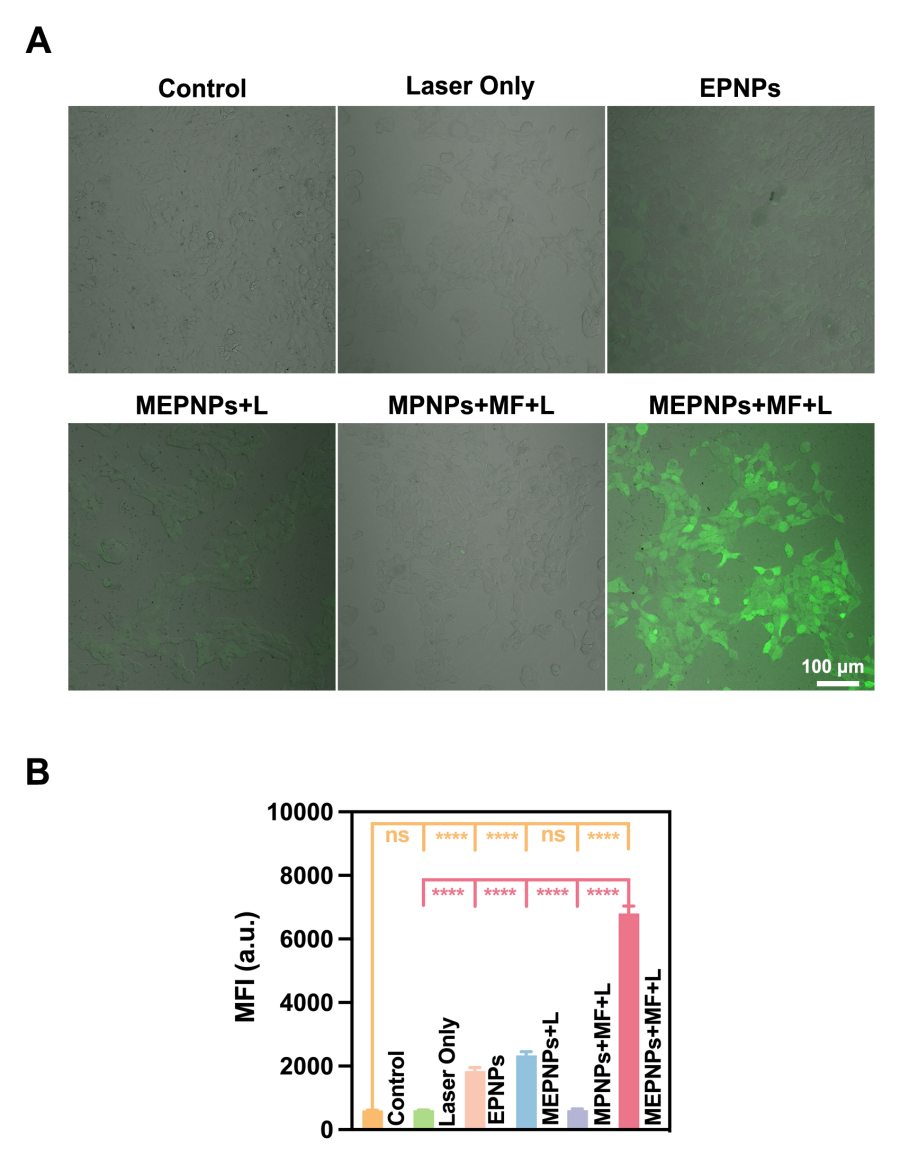
**

**Fig. S7. (A) Observe the production of reactive oxygen species (ROS) after different treatments under CLSM. (B) Detect the average fluorescence intensity of ROS in each group by FCM.**

**
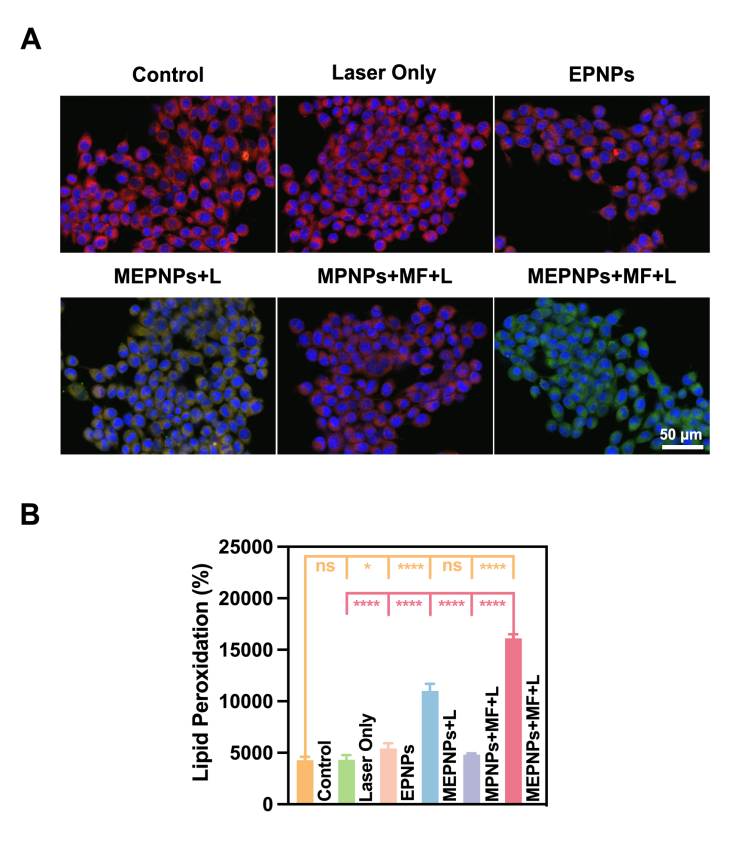
**

**Fig. S8. (A) Observe the degree of lipid peroxidation in different treatment groups under CLSM. (B) Detect the proportion of lipid peroxidation in each group by FCM.**

**
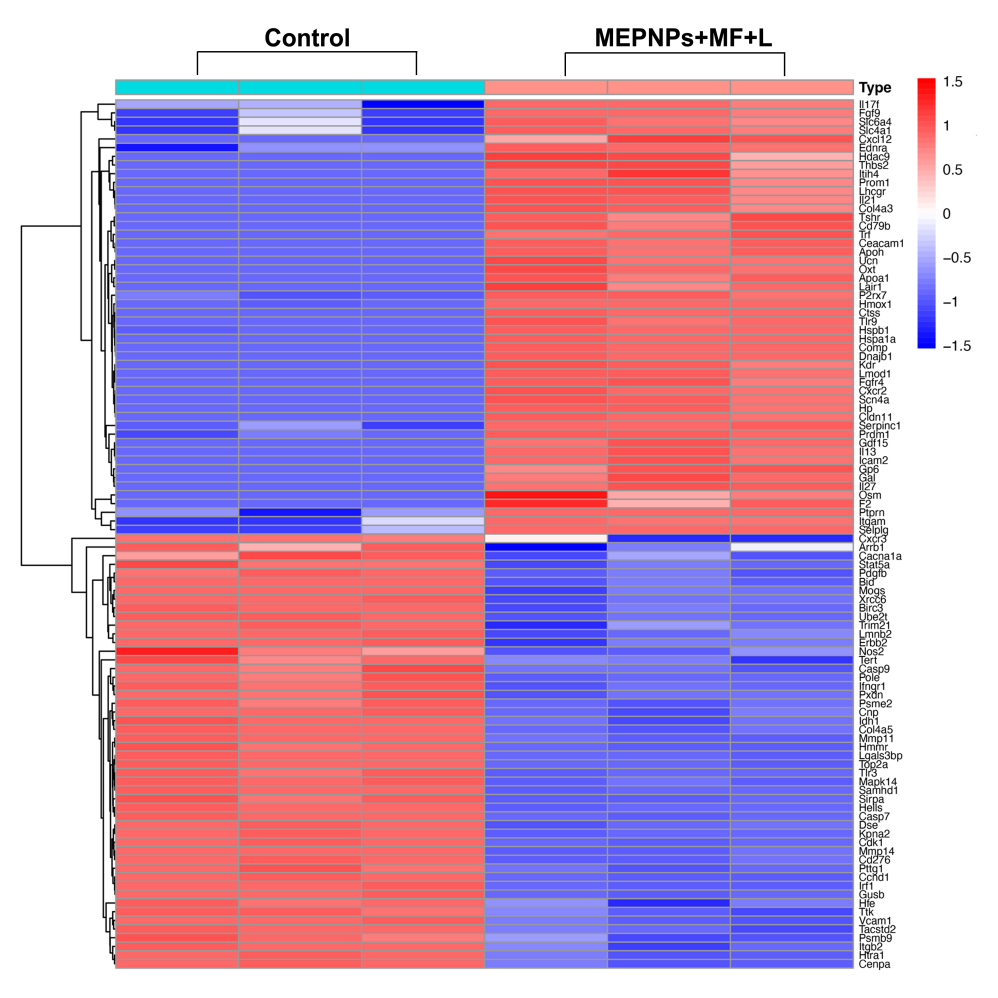
**

**Fig. S9.** **Heatmap of the top 50 ICD-related genes that were upregulated and downregulated after treatment with MEPNPs.**

**
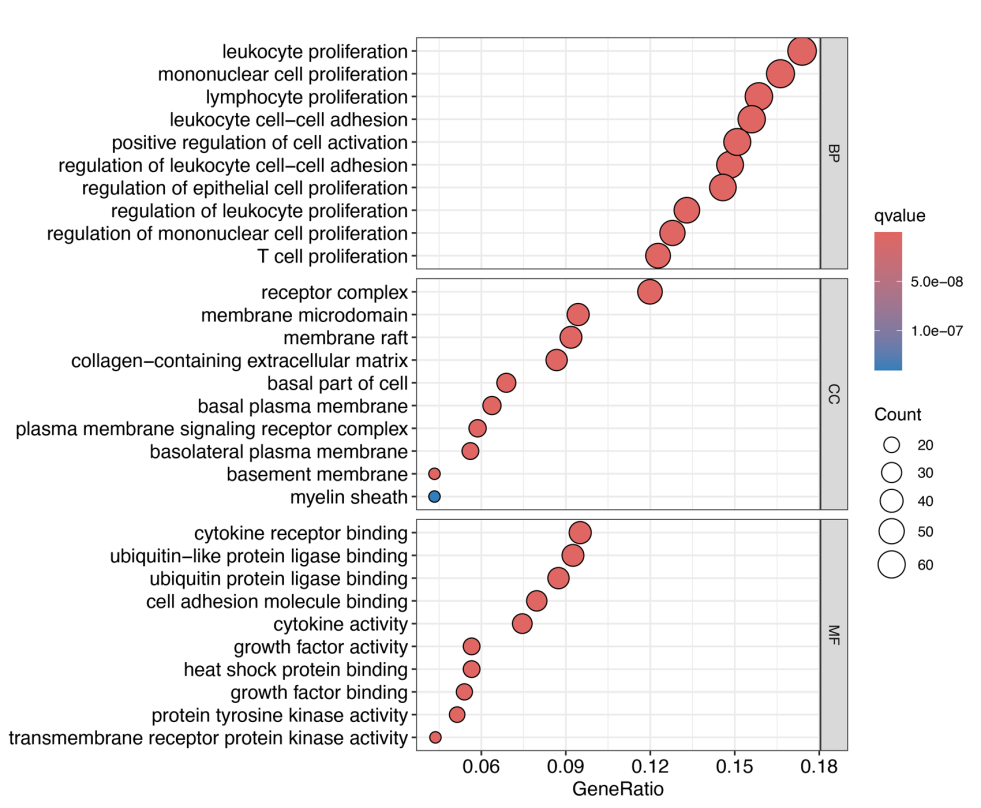
**

**Fig. S10. Bubble plot of GO enrichment analysis of ICD-related differentially expressed genes**

**
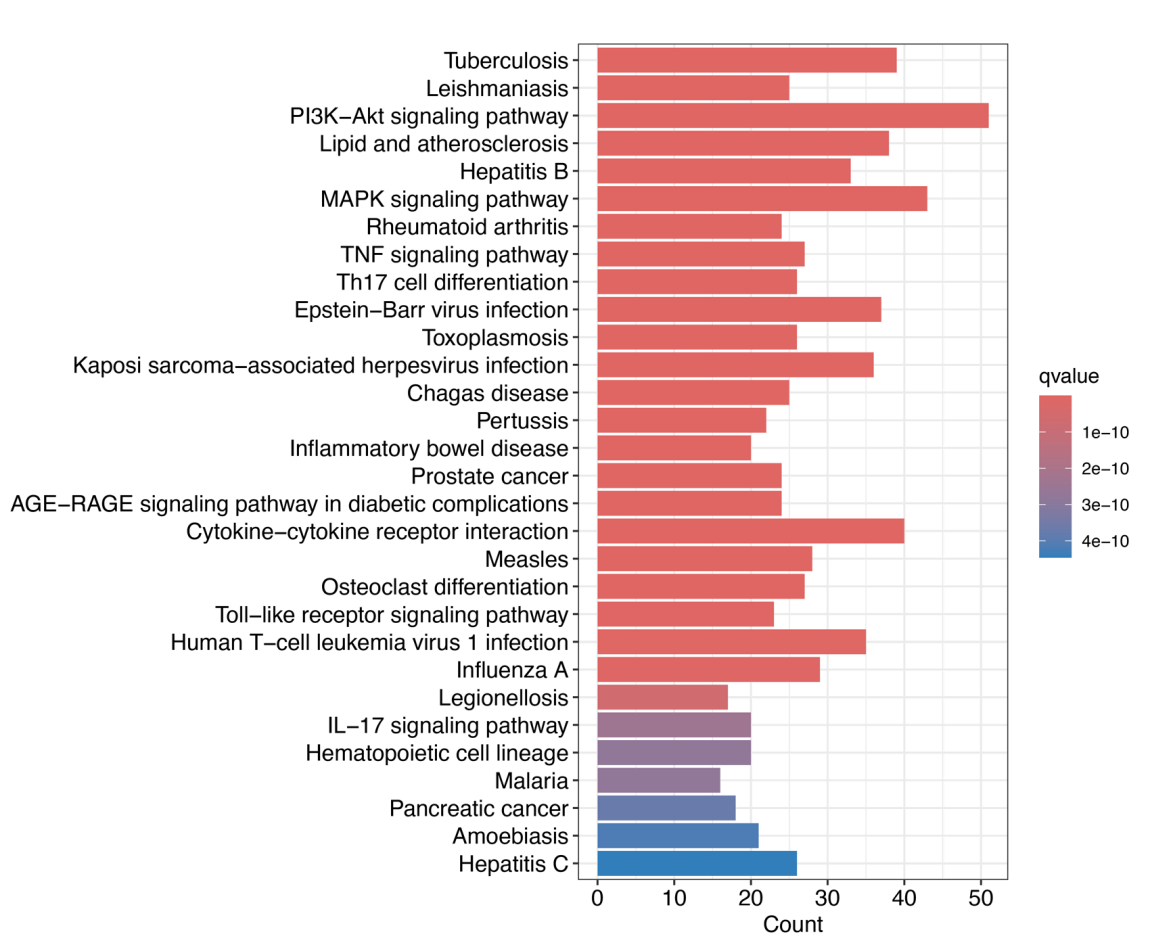
**

**Fig. S11. Bar chart of KEGG enrichment analysis of ICD-related differentially expressed genes**

**
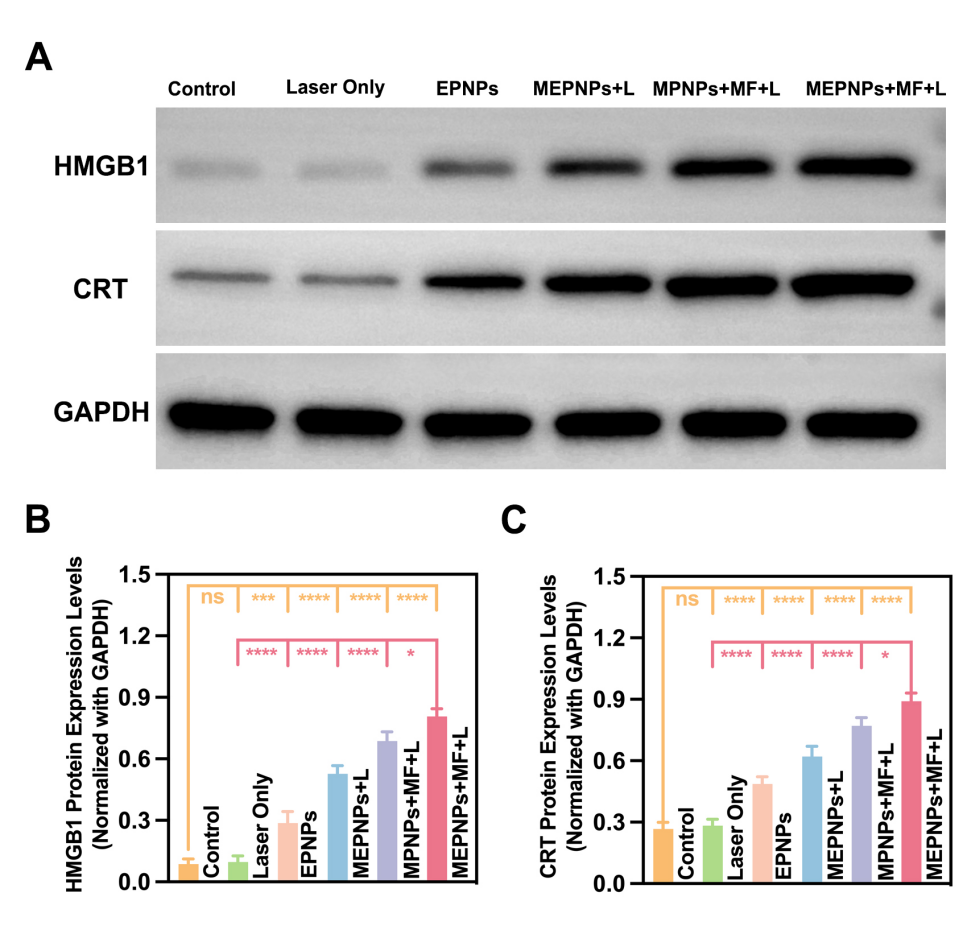
**

**Fig. S12. The expression levels of HMGB1 and CRT proteins were detected by Western blotting**


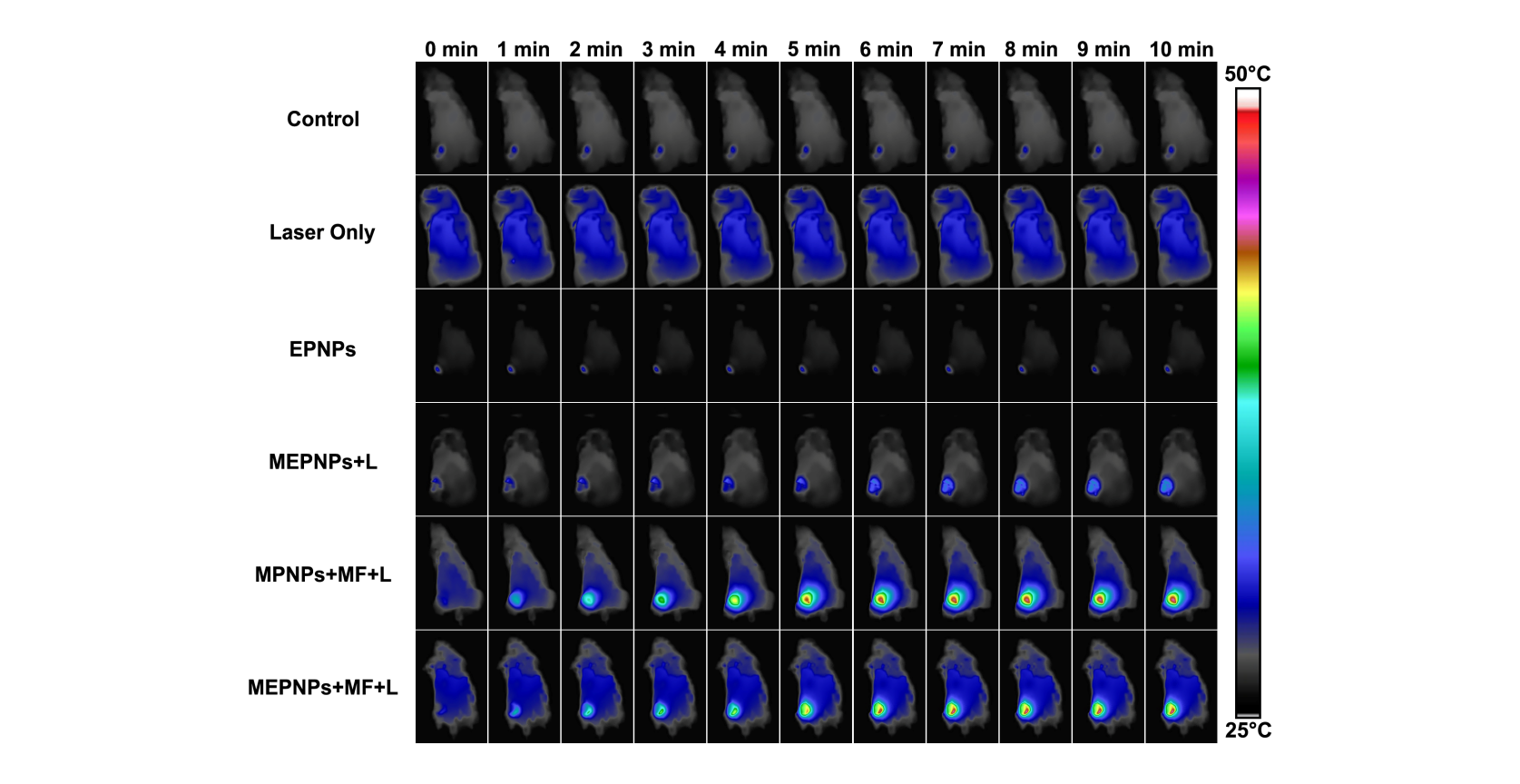


**Fig. S13. Infrared thermal images of mice in each group during the treatment process.**

**
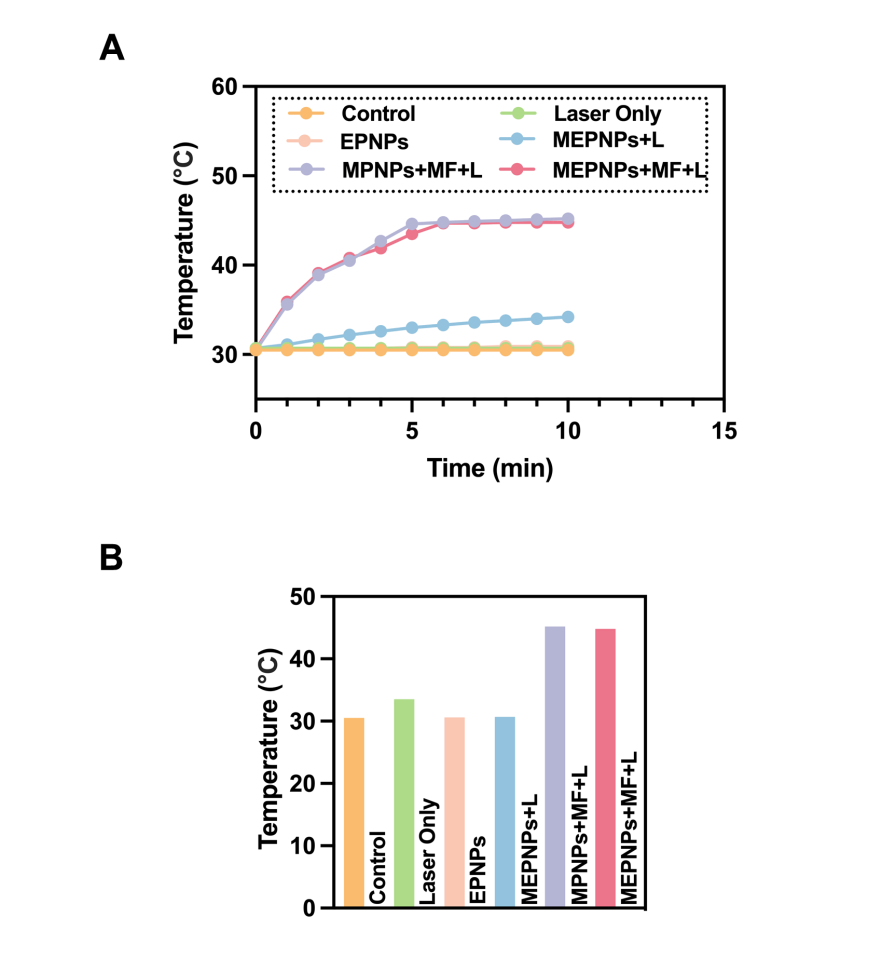
**

**Fig. S14. (A) Temperature change curves of tumor sites in each group. (B) Maximum temperature values of tumor sites in each group.**

**
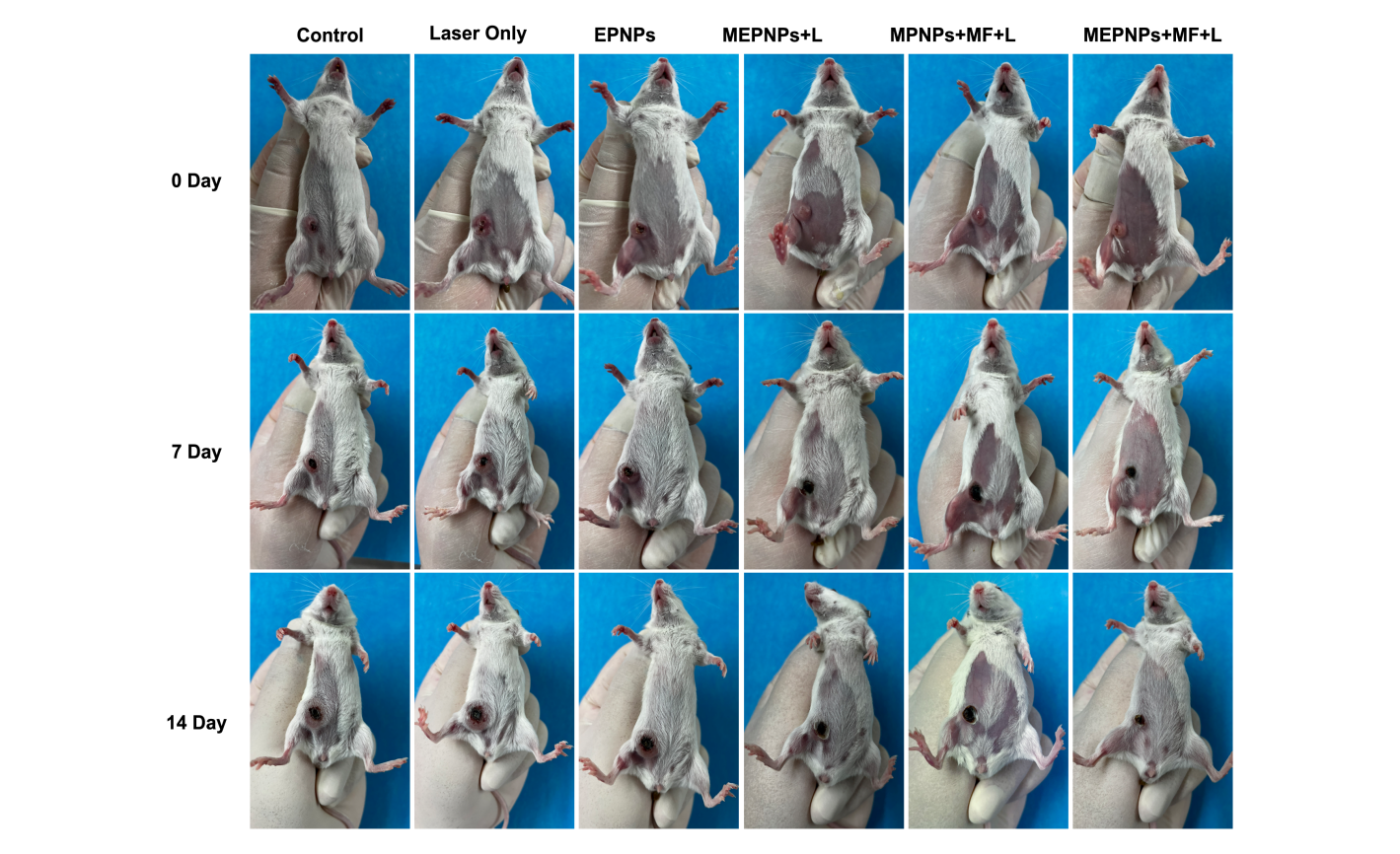
**

**Fig. S15. Digital photos of representative mice in each group during the treatment process.**

**
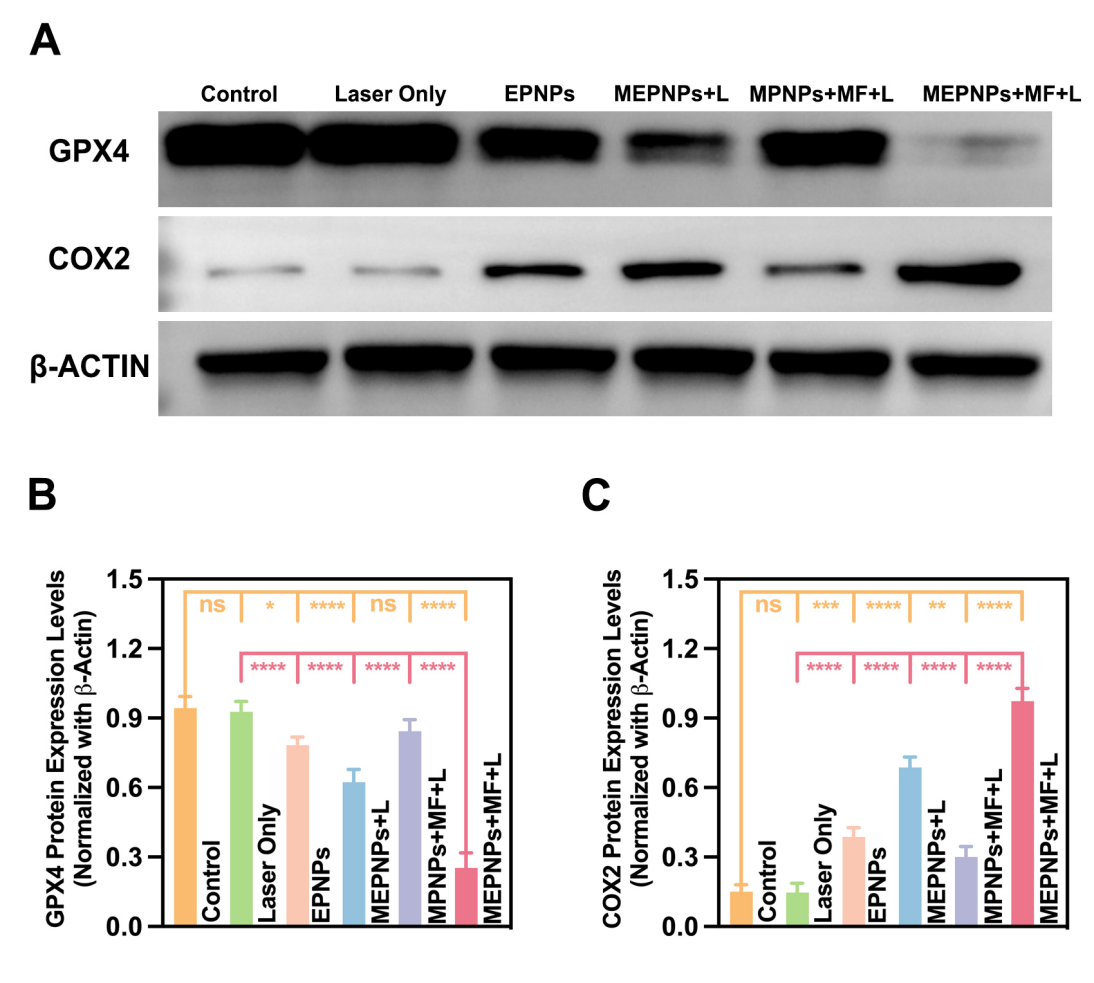
**

**Fig. S16.** **The expression levels of GPX4 and COX2 proteins in tumor tissues were detected by Western blotting**

**
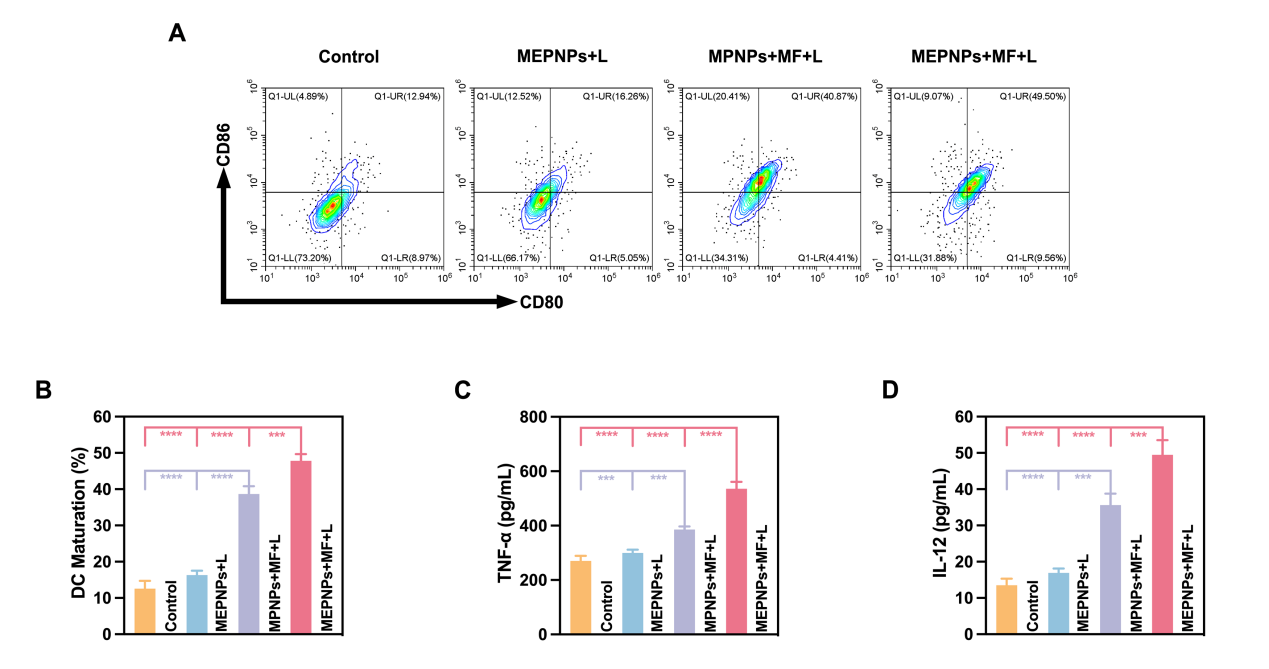
**

**Fig. S17. (A) FCM analysis of the mature proportion of dendritic cells after different treatments. (B) Histogram of the mature proportion of dendritic cells. (C) ELISA detection of TNF-α secretion levels in each group. (D) ELISA detection of IL-12 secretion levels in each group.**

**
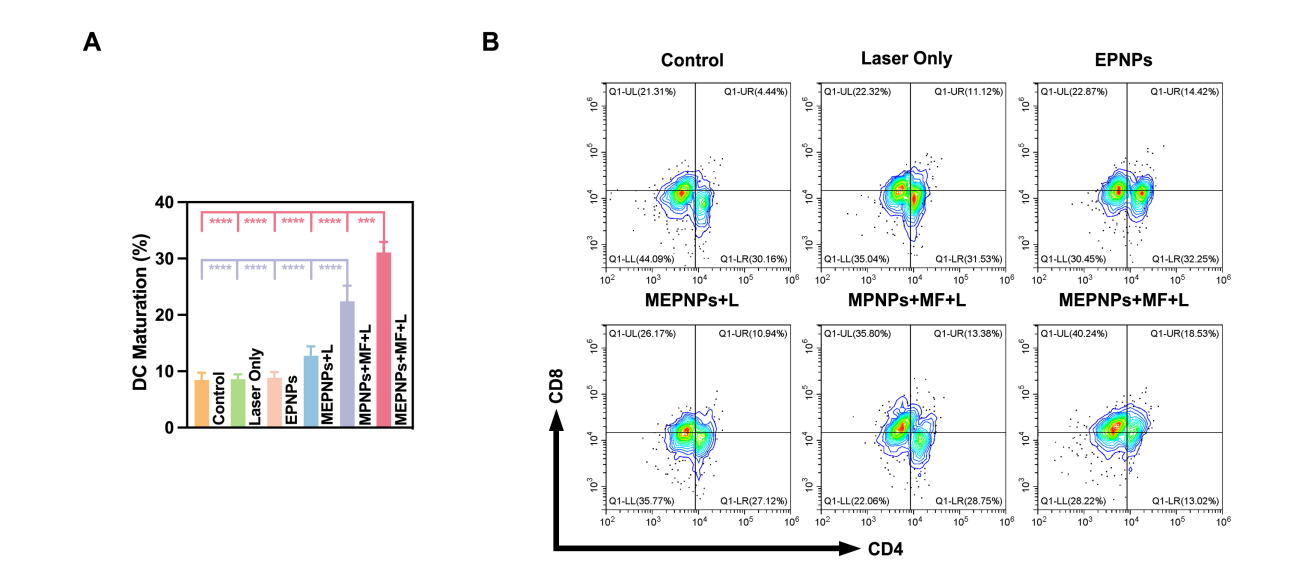
**

**Fig. S18.** **The proportion of mature dendritic cells (DCs) in spleens (A) and CD8^+^ T cells in**  **lymph nodes (B) was detected by FCM.**
